# Sofosbuvir Polymorphs Distinguished by Linearly and Circularly Polarized Raman Microscopy

**DOI:** 10.1021/acs.analchem.4c03573

**Published:** 2024-11-21

**Authors:** Věra Schrenková, Josef Kapitán, Petr Bouř, Argyro Chatziadi, Adam Sklenář, Jakub Kaminský

**Affiliations:** † Institute of Organic Chemistry and Biochemistry of the Academy of Sciences, Flemingovo Nám. 2, Prague 16610, Czech Republic; ‡ University of Chemistry and Technology Prague, Technická 5, Prague 16628, Czech Republic; § Palacký University Olomouc, 17. Listopadu 12, Olomouc 77146, Czech Republic

## Abstract

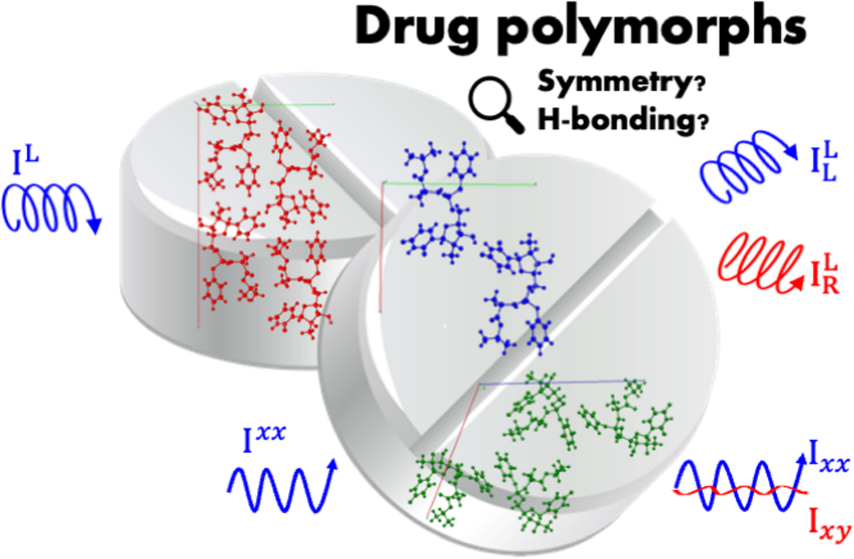

Most currently marketed pharmaceuticals are manufactured in the solid state, where the bioavailability of the active pharmaceutical ingredient (API) can be optimized through different polymorphs, cocrystals, solvates, or salts. Efficient techniques are needed to monitor the structure of pharmaceuticals during production. Here, we explore the potential of linearly and circularly polarized Raman microscopy for distinguishing three polymorphs of sofosbuvir, an antiviral drug used to treat hepatitis C. Raman spectra were recorded on a Raman microscope for a polycrystalline API diluted in a KBr matrix. To understand spectral features including the low-frequency region, we simulated band frequencies and intensities using quantum-chemical computational strategies based on cluster and transfer approaches. Very good agreement was achieved between simulated and experimental intensities. The 20 to 200 cm^–1^ wavenumber region appeared particularly useful for polymorph discrimination already based on unpolarized measurements. The depolarization ratios obtained from linearly polarized Raman spectra made the distinction even more reliable. Moreover, circularly polarized Raman spectra and normalized degrees of circularity provided useful additional information and revealed several tentative markers of the different polymorphs of sofosbuvir. Although in some spectral regions the differences were less obvious, the results indicate that polarized Raman microscopy is a handy tool for discriminating between polymorphs of APIs and other compounds.

## Introduction

Most marketed drugs are formulated in the solid state, but their poor water solubility often limits their bioavailability and therapeutic efficiency.^1^ Formulations such as cocrystals,^2^ salts,^3^ solvates^4^ or polymorphs,^5^ all allow fine-tuning of physical and chemical properties of an active pharmaceutical ingredient (API).^6−8^ Crystal polymorphism, where a compound can adopt multiple crystal structures, is reported for more than half of solid drugs,^5,9^ reliable and reasonably fast analytical methods for structural monitoring are needed to ensure proper product functionality.

Most often, diffraction techniques are used,^10^ followed by solid-state nuclear magnetic resonance^11,12^ and thermal methods.^13^ Vibrational techniques such as Raman^14−16^ and infrared^17^ spectroscopy offer a promising alternative due to their nondestructive nature and sensitivity to differences in crystalline arrangement. A chiral variant of infrared absorption, vibrational circular dichroism, has also proven capable of distinguishing between polymorphs.^18−20^ Also, coupling Raman spectroscopy with confocal microscopy appears convenient because it makes it possible to visualize the distribution of APIs within tablets,^21^ monitoring drug release,^22^ and tracking changes during pharmaceutical production.^23^

With the recent availability of efficient optical filters, the low-frequency (LF) region (sometimes referred to as the terahertz region) has become commonly available on commercial spectrometers and Raman microscopes.^24,25^ LF Raman bands (<200 cm^–1^) are particularly sensitive to intermolecular interactions and therefore closely reflect crystal packing.^26^ It has been shown that LF Raman spectra effectively distinguish drug polymorphs,^15,27,28^ or racemic crystals from enantiopure crystals,^29^ cocrystals^30^ and pharmaceutical excipients.^31^

Compared to unpolarized Raman experiments, additional information on the symmetry of vibrational modes and orientation of crystalline materials can be obtained by controlling the polarization states of incident and scattered light.^32−34^ Although recent advances allow for the simultaneous acquisition of all polarization states,^35^ in our present study we performed separate polarization measurements at each scanning point.

Still, there are relatively few studies that use polarized Raman spectroscopy methods, especially in pharmaceutical applications. In linearly polarized Raman spectroscopy (LPRS), the incident light is linearly polarized, and a parallel or perpendicular component of the scattered light passes through the analyzer. LPRS has been used to study the crystallographic orientation of single crystals,^36^ 2D materials^37^ and nanowires.^38^ Also, LPRS can be used to ascertain the preferential orientation of API particles on the surface of tablets^35,39^ or different intermolecular hydrogen bonding over a wide temperature range in paracetamol polymorphs.^40^

Similarly, in circularly polarized Raman spectroscopy (CPRS), incident and scattered light is circularly polarized, either in the same or opposite helicity.^41^ CPRS has been applied, in particular, to investigate lattice properties of layered materials^42^ and to determine the axial angle in chiral nanotubes.^43^ As far as we know, no application of CPRS to solid-phase pharmaceutical samples has hitherto been reported.

Strictly speaking, CPRS does not provide additional information compared to LPRS, as the same molecular properties are involved.^44^ However, the practical advantages of the two approaches remain to be ascertained. For example, differences between polymorphs may be better visible in CPRS, or experimental artifacts might be smaller with CPRS than with LPRS. Either polarization technique can enhance spectral resolution by resolving bands that overlap in nonpolarized experiments.^45^

Alongside experimental advancements, computational techniques have greatly improved the interpretation of both polarized and unpolarized Raman spectra in both solutions and solids.^46−49^ Plane–wave (PW) density functional theory (DFT) is often applied to crystals, as demonstrated for ribavirin polymorphs.^50^ Here, we use PWDFT^51^ only to improve X-ray geometries, because the method appears to be too slow for studying the vibrational properties of large molecules. Therefore, we combine or replace PW computations with cluster-based and Gaussian basis set methods. Because of the size of sofosbuvir, we use Cartesian coordinate tensor transfer (CCT)^52^ to calculate important intermolecular interactions taking place within crystals. Earlier, this approach was used to calculate Raman spectra of crystalline and amorphous poly(3-hydroxybutyrate),^53^ as well as Raman spectra of methacrylamide, piracetam and 2-thiobarbituric acid polymorphs.^54^

**Figure 1:**
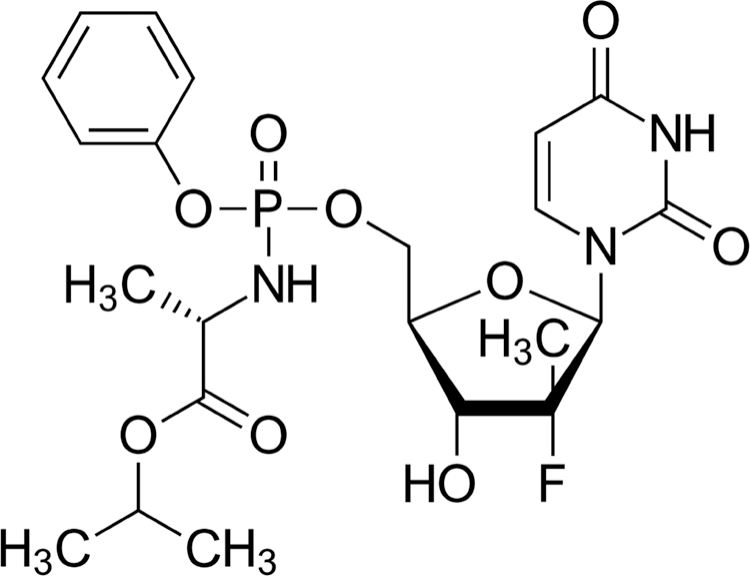
Structure of sofosbuvir.

Below, we present Raman spectra (unpolarized, LPRS and CPRS) of three polymorphs of the antiviral drug sofosbuvir across a broad spectral range (20–3500 cm^–1^). Sofosbuvir (Figure 1) is used to treat chronic hepatitis C (HCV) because it inhibits NS5B nucleotide polymerase.^55^ Sofosbuvir’s flexibility related to the phosphate group contributes to its rich polymorphism. At least 15 polymorphs of sofosbuvir have been reported so far,^56^ along with its cocrystals or solvates.^57^ The spectra of the polymorphs studied here are recorded for a polycrystalline API pressed into pellets, eliminating the need for single crystals. DFT simulations then allow us to more accurately identify relationships between the structure of compounds and their spectra. This study demonstrates the potential of polarized Raman microscopy for the precise characterization of pharmaceutical polymorphs, providing a valuable tool for drug development.

## Methods

### Sofosbuvir Structure

**Figure 2:**
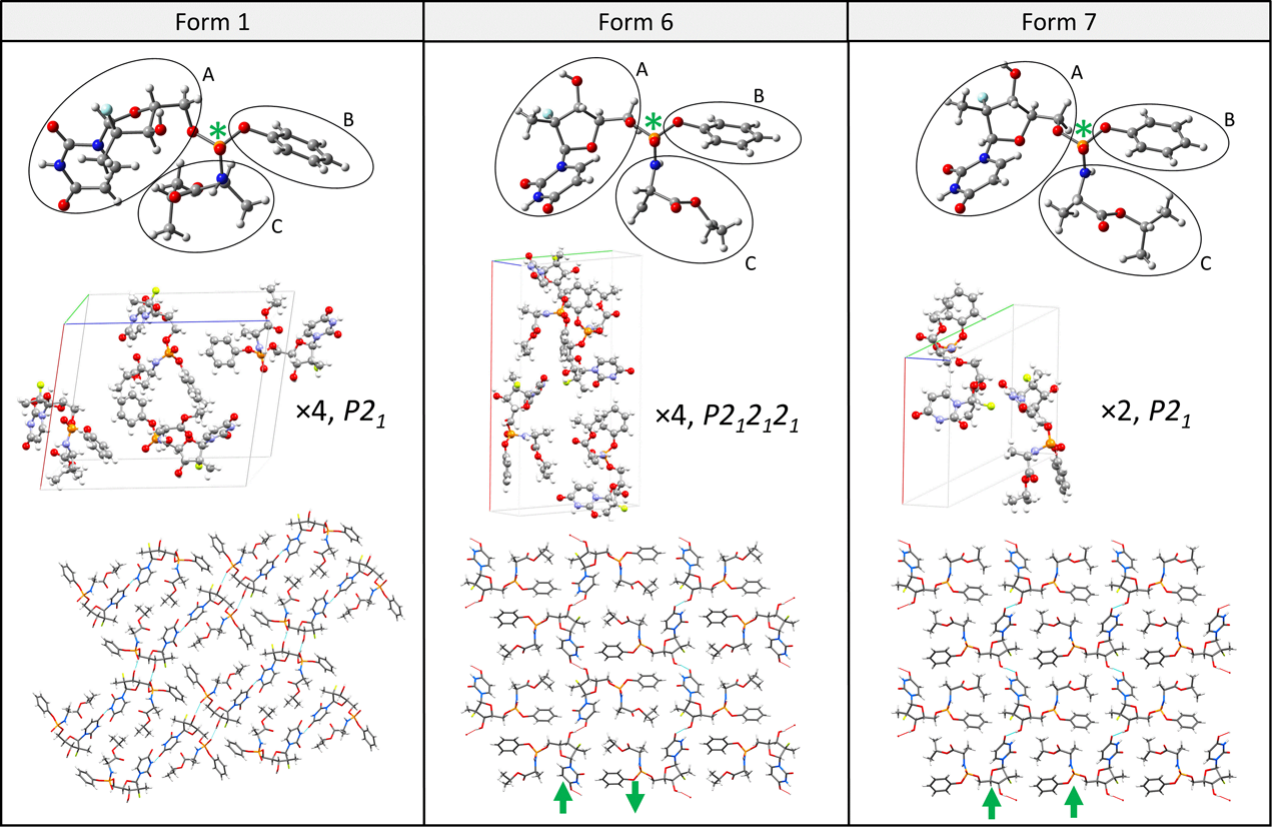
Single molecules, elementary cells and larger parts with H-bonded chains of sofosbuvir polymorphs under study,^58^ denoted as form 1, form 6 and form 7. A = nucleotide group, B = phenyl group, C = l-alanine isopropylester group. The phosphorus atom is marked with an asterisk (*) for better orientation.

Polymorphs chosen for this study are denoted as form 1, form 6 and form 7 (Figure 2), and they were prepared by Chatziadi et al.^58^ Form 1 crystallizes in a monoclinic crystal lattice with the *P*2_1_ space group and comprises four molecules in the elementary cell. Form 7 also crystallizes in a monoclinic lattice (space group *P*2_1_), but it contains two molecules in the elementary cell.

Form 6 is the most compact and stable at room temperature, adopting an orthorhombic crystal lattice with the *P*2_1_2_1_2_1_ space group, and there are four molecules in the elementary cell. The conformation of sofosbuvir in form 1 differs significantly from that in the others. Forms 6 and 7 are conformationally similar, as they differ mainly in the orientation of the l-alanine isopropyl ester group (marked as C in Figure 2). Forms 6 and 7 create hydrogen-bonded chains that link similar sofosbuvir rotamers. In form 7, the hydrogen bond chains are oriented in the same direction whereas in form 6 they alternate (i.e., are flipped by 180°). All three forms have been recently studied using vibrational circular dichroism.^20^

### LPRS and CPRS Parameters

In LPRS, the ratio of perpendicular (*I*_⊥_) to parallel (*I*_∥_) intensities with respect to the polarization plane of the incident light is called the depolarization ratio ρ = *I*_⊥_/*I*_∥_. For isotropic samples it can be related to molecular parameters as
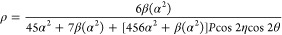
where α^2^ and β(α^2^) are invariants of the polarizability tensor and *P*, θ and η describe the polarization and geometry of the experiment. Parameter ρ reflects the symmetry of vibrational modes; totally symmetric modes give β(α^2^) = 0 and ρ = 0 whereas asymmetric modes give α^2^ = 0 and ρ can reach 3/4, the maximum value for a backscattering experiment.^25^ The value of ρ is generally nonzero.

In analogy to ρ, the degree of circularity (DOC) is defined in CPRS as
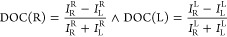
where the upper and the lower index denotes the helicity of the incident or scattered light, respectively. For a backward scattering geometry (180°), it can be calculated from as
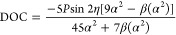
where *P* = 1 and  for right- or left-handed circularly polarized incident light. The scattered light completely reverses circular polarization for β(α^2^) = 0 (DOC = −1), and it becomes partially circularly polarized () in the same sense if α^2^ = 0; DOC of fluorescence signal is close to zero (apart from fluorescence-detected CD signal, CPL, etc.). Under backscattering conditions, one can convert DOC to ρ, for example to verify experimental CPRS results, as
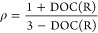


### Sample Preparation

Three polycrystalline polymorphs of sofosbuvir (form 1, form 6 and form 7) were prepared according to previously reported protocols.^58^ The purity of the polymorphs was confirmed by measuring their melting point temperatures (form 1 = 96 °C, form 6 = 118 °C, form 7 = 125 °C), which were consistent with previously published values.^20,58^ Raman maps were recorded for polycrystalline sofosbuvir in KBr. This matrix is regularly used in infrared spectroscopy and appeared convenient also for Raman experiments. It enabled us to homogenize samples and reduce polarization artifacts. Also, KBr to some degree mimics the environment within medicinal tablets. Sofosbuvir and potassium bromide (Sigma-Aldrich, ≥97%) were finely ground together in an agate mortar in a 1:1 ratio. The resulting mixture was compressed into a thin transparent pellet (7 mm in diameter) using a laboratory hydraulic press (Specac Mini Pellet Press). Following ref (20), polymorphic purity was additionally checked by *k*-means cluster analysis of the spectra obtained from the maps, which confirmed the occurrence of only one form in the sample.

### Data Acquisition

**Figure 3:**
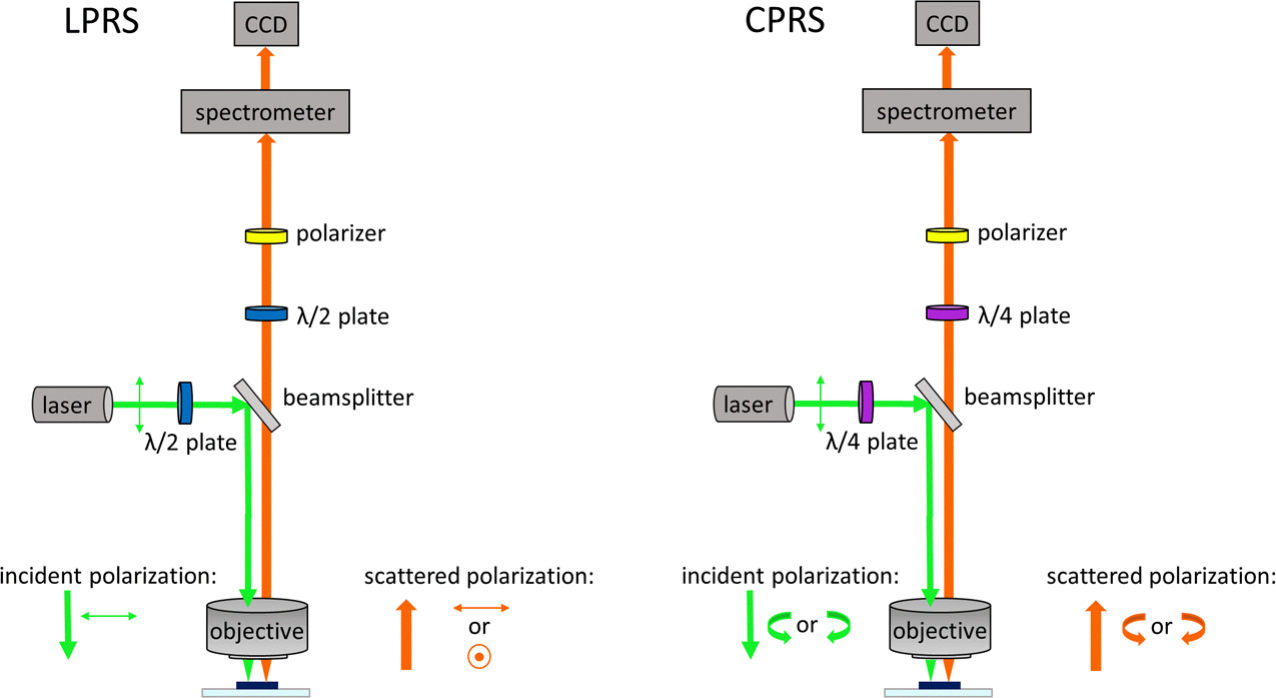
Raman microscope setup for linearly (left) and circularly (right) polarized modes. Unpolarized (*I*_u_), parallel (*I*_∥_) and perpendicular (*I*_⊥_) spectra were recorded in the LPRS mode, corotating (*I*_L_^L^, *I*_R_^R^) and contrarotating (*I*_R_^L^, *I*_L_^R^) spectra in the CPRS mode.

Raman experiments were carried out using two setups of the WITec Alpha 300RS confocal Raman microscope (Figure 3).

We recorded a spectral map for each polymorph in each optical arrangement (parallel and perpendicular setup for LPRS, corotating and contrarotating setup for CPRS). For LPRS, the microscope was equipped with two half-wave plates placed in front and behind the sample, and the light was analyzed using a polarizer. For CPRS, we used a quarter-wave plate to convert linearly polarized light coming from the laser into circularly polarized light. The scattered light then passed through a second quarter-wave plate and the polarizer. Raman mapping of the pellets was performed in a backscattering geometry. A 10× dry objective lens (Zeiss EC Epiplan, numerical aperture 0.25) was used to focus the laser beam onto the sample and to collect the scattered light. A laser with a wavelength of 532 nm and a power of 19 mW, with 1800 g/mm diffraction grating for spectra below 200 cm^–1^ and 600 g/mm grating for spectra at and above 200 cm^–1^, was used. The Raman mapping was done for an area of 300 × 300 at 2 μm steps (150 × 150 points) with 1 s integration time. Each image consisted of 22,500 spectra.

### Data Processing

Raman maps were preprocessed using WITec Project 5.1 software. First, we applied a cosmic ray removal filter (filter size 4, dynamic factor 5) and then we averaged the signal over all pixels. Because Raman bands are narrower than other signal components, the baseline was subtracted from the spectra using our own implementation of the rubberband algorithm.^59^

### Spectral Simulations

Spectra of all sofosbuvir polymorphs were calculated at the DFT level for structures derived from X-ray geometries (CCDC entries CUZROG01, CUZROG02 and CUZROG03) using the fragmentation and tensor transfer approach, which has been successfully used to model the vibrational spectra of various compounds in the condensed^20^ or semicondensed state.^60^ All details of the procedures can be found in the Supporting Information.

Because the positions of hydrogen atoms determined by X-ray diffraction may not be exact, we used three computational strategies to refine our geometries. In the first approach, we optimized the geometry of the unit cell using the PW basis sets technique as implemented in the program CASTEP^51^ before assembling the 3 × 3 × 3 fragment. Alternatively, we partially optimized only the molecular pairs detected and created from the fragment, using the QGRAD program^61^ interfaced to Gaussian 16 (ref (62)) in normal mode (NM) vibrational coordinates.^63^ In the third, “combined” approach, we preoptimized the crystal unit cell in CASTEP and then optimized the molecular pairs using the NM coordinates.

The harmonic force field and polarizability derivatives were then calculated for the optimized molecular pairs, using the program Gaussian^62^ at the B3LYP/6-311++G(d,p) level. The crystal environment was simulated using the CPCM solvent model^64^ and relative permittivity ε_r_ = 78.^54^ Subsequently, the atomic property tensors were transferred back to the 3 × 3 × 3 fragment, using the CCT scheme.^52^ Vibrational frequencies and Raman intensities were calculated in two ways: (1) directly for the 3 × 3 × 3 cluster and (2) using a dynamic matrix constructed from the same force field and the zero (0, 0, 0) crystal phonon mode. Line intensities, depolarization and DOC ratios were convoluted with Lorentzian bands of 10 cm^–1^ full width at half height. Frequencies are not scaled, and integral intensities were normalized against experimental values within the 200–1800 cm^–1^ range.

## Results and Discussion

### Theoretical Models

**Figure 4:**
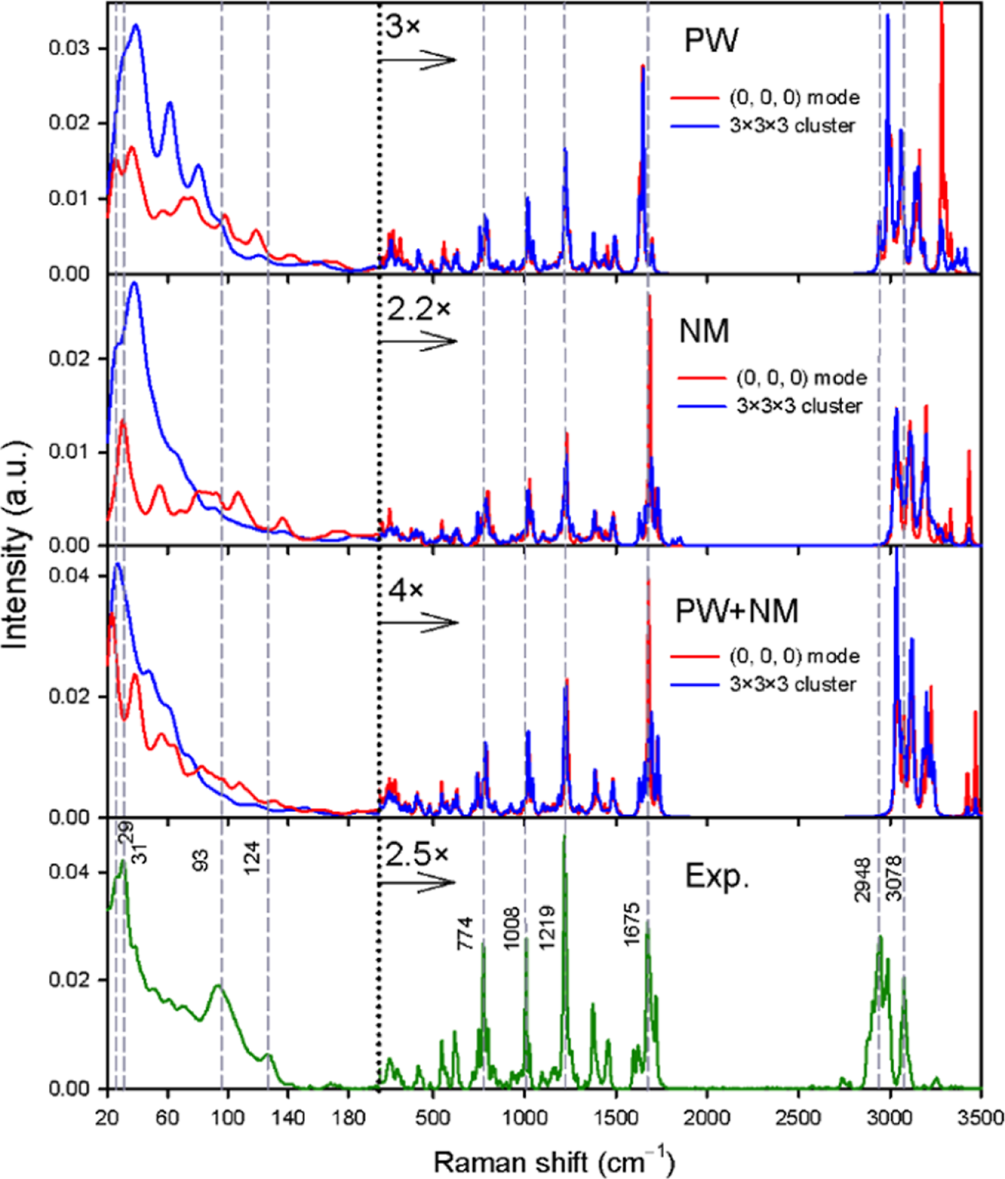
Comparison between calculated unpolarized Raman spectra of sofosbuvir form 7 and experimental data. The B3LYP/6-311++G(d,p)/CPCM level was used. Frequencies are not scaled and integral intensity is normalized against experimental values within the 200–1800 cm^–1^ range.

Figure 4 shows the effect of geometry optimization and vibrational modeling on calculated unpolarized Raman spectra of form 7, in comparison with experimental data. The optimization strategies included the PW, NM optimization and a combined approach (see the Methods section). The calculated and experimental frequencies of the strongest bands are compared in more detail in Figure SI and Table 1.

**Table 1:** Experimental and Calculated Frequencies of Selected Raman Bands of Sofosbuvir Form 7

	PW optimization		NM optimization		PW + NM optimization	
exp.	3 × 3 × 3 crystal	dynamic cell	3 × 3 × 3 crystal	dynamic cell	3 × 3 × 3 crystal	dynamic cell
29	30	26	26		27	23
31	39	36	38	30		38
	61	71		54	47	56
93	80	98		81		82
124		119		107		
774	780	786	784	799	786	792
1008	1018	1019	1019	1027	1020	1022
1219	1222	1220	1232	1230	1224	1231
1675	1649	1648	1694	1649	1695	1678
2948	2988	2988	3042	3036	3036	3040
3078	3278	3284	3195	3198	3201	3223

Although the main spectral features are well reproduced by all three optimization approaches, in the lowest-wavenumber region the spectral shapes strongly depend on fine differences in the optimization. PW optimization inherently includes long-range interactions in the crystal. However, employing a different DFT level, a different description of a basis set for the subsequent calculation of vibrational properties may introduce errors. On the other hand, NM optimization is performed at the same theoretical level as the force field, but only molecular pairs without long-range interactions are considered in the calculation. The calculated frequencies correspond closely to equilibria on the potential energy surface, particularly for modes that were not constrained. The combined approach may compensate for some of the disadvantages of the two approaches.

The bands within the ∼20 to 200 cm^–1^ range result from modes involving translations/rotations of various molecular parts and whole crystal layers. This spectral part is better reproduced by the zero-crystal phonon model, which otherwise gives results almost indistinguishable from those obtained for the 3 × 3 × 3 cluster. The “3 × 3 × 3” spectra below 200 cm^–1^ are less structured than in experimental data. Supposedly, the bands at 30, 93, and 124 cm^–1^, calculated using the PW method, correspond to experimental signals at 36, 98, and 119 cm^–1^. All calculations predict a large intensity below 200 cm^–1^.

In the fingerprint region (∼200 to 1800 cm^–1^), the PW method was in slightly worse agreement with experimental data than the others. This is apparent for the C=N stretching and NH_2_ bending vibrational pattern, experimentally determined to occur at around 1675 cm^–1^, where the PW method produces an unrealistically sharp band at 1649 cm^–1^. However, given its good performance with LF Raman spectra, this is only a minor drawback of PW optimization, which can still be recommended as a universal approach to first-principles calculations.

Within the 2800–3500 cm^–1^ range, all calculated frequencies were overestimated, mostly because the anharmonic effects were neglected.^54,65^ The relative intensity of out-of-phase and in-phase NH bands (>3200 cm^–1^) is often overestimated by calculations.

### Recognition of Polymorphs in the LF Region

**Figure 5:**
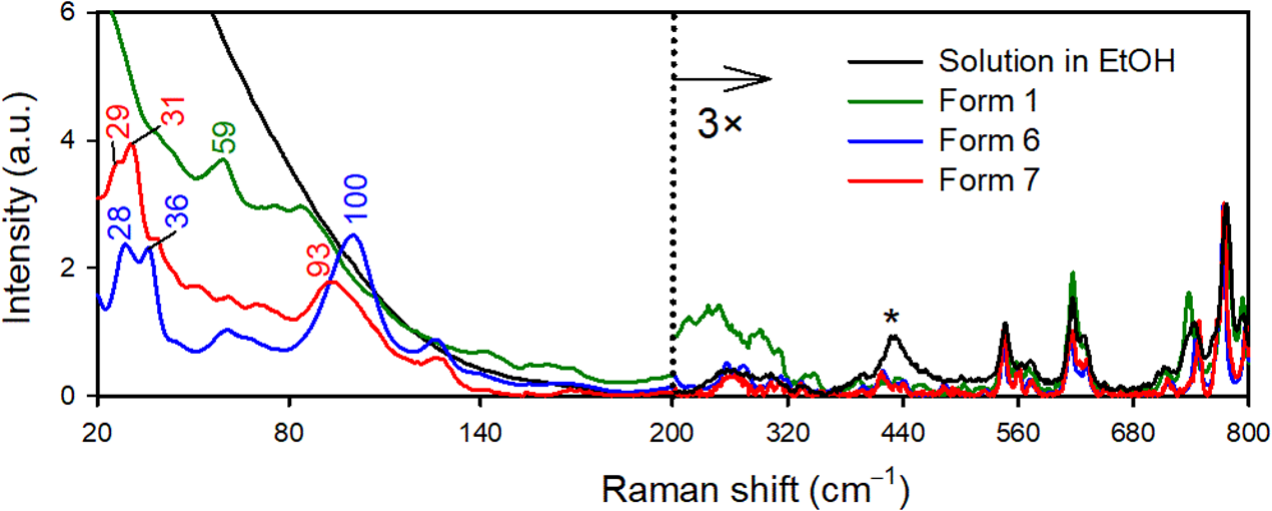
Unpolarized experimental Raman spectra of solid state sofosbuvir forms 1, 6, and 7 and of a 100 mg/mL solution of sofosbuvir dissolved in ethanol (the asterisk marks the ethanol band).

Whereas only minor differences among the polymorphs were observed within 200–3500 cm^–1^ range (Figure SII), larger variations occur below 200 cm^–1^ (Figure 5). In Figure 5, we also included the spectrum of sofosbuvir dissolved in ethanol, to document the better-resolved bands in the solid state, which are sensitive to crystal packing.^66^

The differences in the spectra of polymorphs in the LF region partially reflect the conformation of sofosbuvir but are influenced to a greater degree by the environment within the crystal and by crystal packing. To demonstrate that, we compared spectra of the conformationally similar polymorphs 6 and 7 calculated for isolated molecules in a vacuum with those obtained for a crystal (see Figure SIII). We can see that the spectra of isolated molecules are similar whereas those of molecule in different crystal arrangements differ much more.

In form 7, the H-bonded chains are oriented in the same direction whereas in form 6 they alternate. The LF Raman spectrum of form 1 notably differs from those of form 6 and 7 (see also Figure SII), likely reflecting the difference in conformation. Crystal packing does not seem so important. The same trend was previously observed for chlorpropamide, forms II and IV of which exhibited similar LF Raman spectra despite having different crystal packing due to the orientation of the aromatic ring between the consecutive molecules.^67^ On the other hand, forms III and IV of chlorpropamide had distinct LF Raman bands owing to conformational polymorphism and the head-to-tail orientation of chlorpropamide molecules.^67^ For carbamazepine polymorphs, it has been proposed that crystals having greater density and higher symmetry produce bands with lower frequencies.^68^ However, no such trend was observed for theophylline polymorphs.^68^ Similarly, for sofosbuvir polymorphs, the density of which decreases from form 6 to form 7 and form 1 (6 > 7 > 1) and whose symmetry is higher in form 6 than in forms 1 and 7 (6 > 1, 7), we observe no correlation between density, symmetry and band frequency. For a better idea, visualizations of the computed LF modes (PW, zero phonon mode) are presented in Figure SIV.

**Figure 6:**
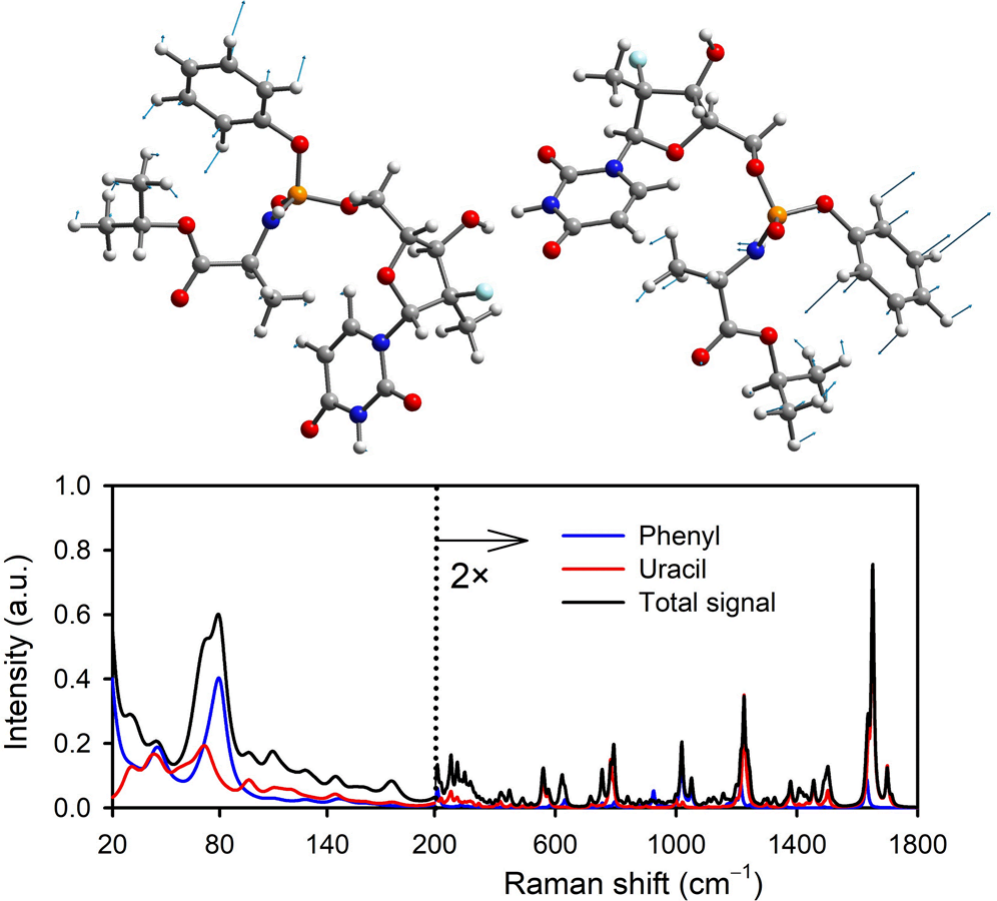
Examples of LF NMs of form 6 of sofosbuvir and the approximate contributions of aromatic rings to the total Raman signal.

The intensity of LF bands is approximately three times greater than that of typical fingerprint bands. This may be explained by the large amplitude of LF vibrations, their temperature-induced excitations and the movement of highly polarizable π-systems.^68,69^ To estimate the contributions originating from the phenyl group and the uracil ring, we set the polarizability derivatives with respect to other atoms to zero. The resulting spectra are plotted in Figure 6. Indeed, we see that the phenyl signal dominates in the LF region. This means that the strongest band at 79 cm^–1^ involves phenyl motion and dimethyl deformation with contributions from the uracil ring and deformation of the methyls in the l-alanine isopropylester group. Of course, the rings, especially the of uracil, also significantly contribute in the fingerprint region.

### Linearly Polarized Raman Spectra

**Figure 7:**
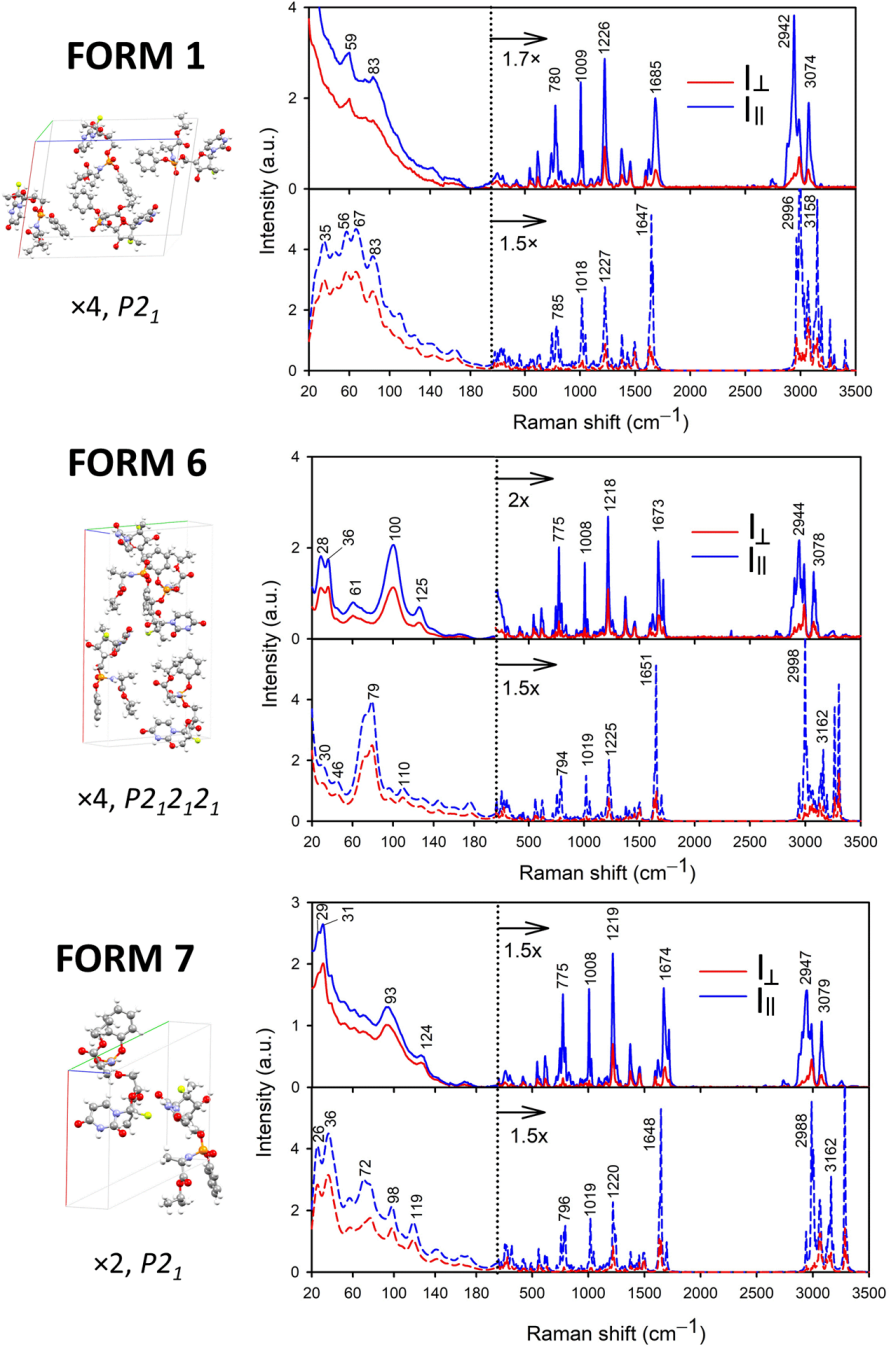
Experimental (upper) and calculated (lower) LPRS spectra of sofosbuvir forms 1, 6, and 7. Intensity is normalized to the ∼775 cm^–1^ band.

Calculated (PW optimization, zero phonon mode) and experimental LPRS spectra of sofosbuvir forms 1, 6, and 7 are provided in Figure 7. The assignment of selected bands and depolarization ratios for the different computational models used are listed in Tables SI–SIV.

In Figure 7, we observe good agreement between calculated and experimental data also for the LPRS spectra. In contrast to the fingerprint region, where the depolarization ratios are rather low, in the LF region these ρ_Exp._ are greater than 0.50. This may reflect the contribution of intermolecular vibrations, which are more complex and more asymmetrical than localized vibrational modes.^26^ Raman signal in the fingerprint region (∼200 to 1800 cm^–1^) involves relatively localized vibrations. The bands are therefore generally less sensitive to the conformation of the molecule and to the environment than LF vibrations. This does not apply to C=O or O–H stretching, which is sensitive to the environmental interactions mediated by hydrogen bonding. Although some differences between the three crystalline forms are observable, they are not as pronounced as in the LF region. In spite of the different crystal packing in forms 6 and 7, their LPRS in the fingerprint region is very close. For example, both form 6 and form 7 have an intense band corresponding to C=N and C=O valence stretching vibrations mixed with N–H deformation found at 1674 cm^–1^ (form 6) or at 1675 cm^–1^ (form 7). For form 1, this peak is shifted to 1685 cm^–1^ and is broader. Analogous similarity between different polymorphs with different packing, but in unpolarized Raman spectra, has been described also for piracetam.^54^

The hydrogen-stretching region (>2800 cm^–1^) appears to be the least convenient for discriminating between the polymorphs, although some differences can be found in this region as well. For the symmetric methyl stretching at ∼2944 cm^–1^, depolarization varies the most (form 1—ρ_Exp._ = 0.08, form 6—ρ_Exp._ = 0.16, form 7—ρ_Exp._ = 0.10). For form 1, the aryl signal occurs at 3074 cm^–1^ whereas it is shifted to 3078 cm^–1^ in form 6 and 3079 cm^–1^ in form 7.

**Figure 8:**
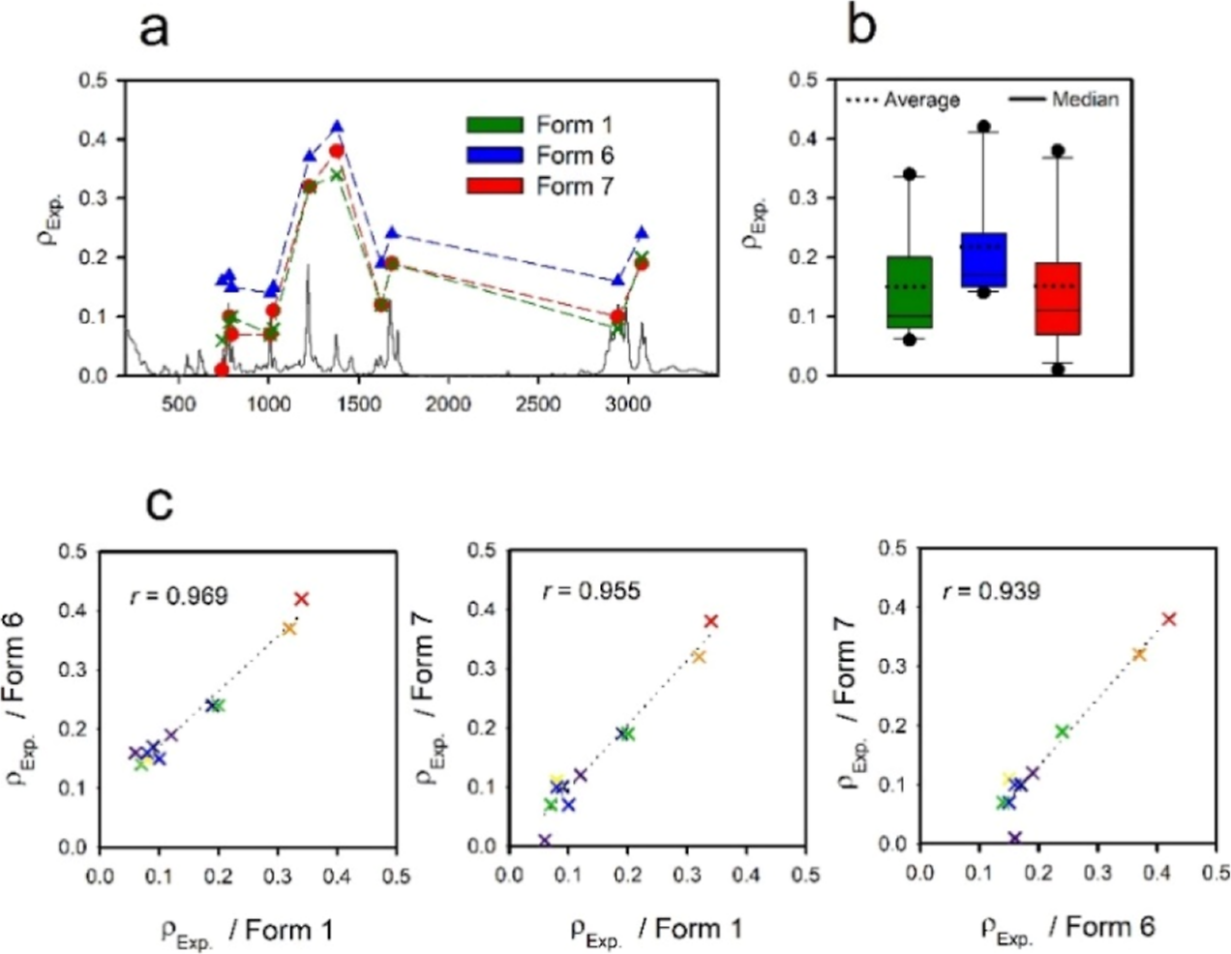
Experimental depolarization ratios (ρ_Exp._) of ten Raman bands with the most significant differences between the three forms of sofosbuvir [panel (a)]. Panel (b) summarizes the distributions of depolarization ratios, panel (c)-correlation coefficients across polymorph pairs.

Because the differences in the fingerprint region and the hydrogen-stretching region (>200 cm^–1^) are less apparent, we analyze them in more detail in Figure 8 (panel a). Form 6 exhibits the highest values of ρ_Exp._, and forms 1 and 7 are similar. Figure 8, panel b summarizes the distributions of ρ_Exp._; the average of form 7 (0.15) is identical to that of form 1, the range is the widest for form 7, and form 6 has the highest average (0.22) and the narrowest range. Nevertheless, the correlation coefficients (Figure 8, panel c) are very high across the polymorph pairs.

**Figure 9:**
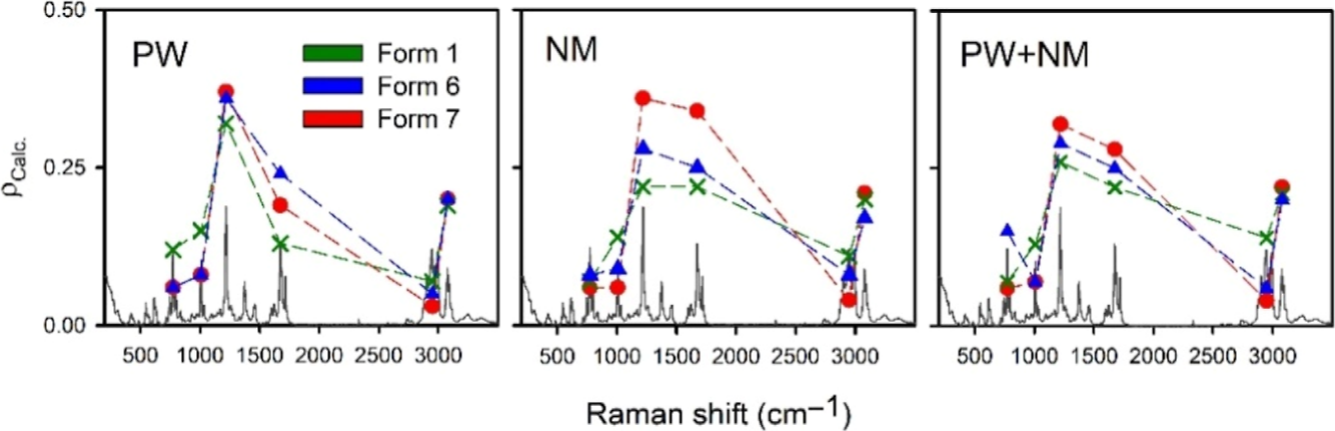
Calculated (0, 0, 0) phonon mode depolarization ratios (ρ_Calc._) of six Raman bands for sofosbuvir form 1, 6, and 7. The plotted Raman spectrum corresponds to form 6.

The calculated depolarization ratios (Figure 9) well correlate with the experimental ones, although the calculations often do not reproduce the fine differences between the polymorphs, presumably because of band overlap and a lack of anharmonic interactions (for the C–H stretching) in the modeling.

### Circularly Polarized Raman Spectra

**Figure 10:**
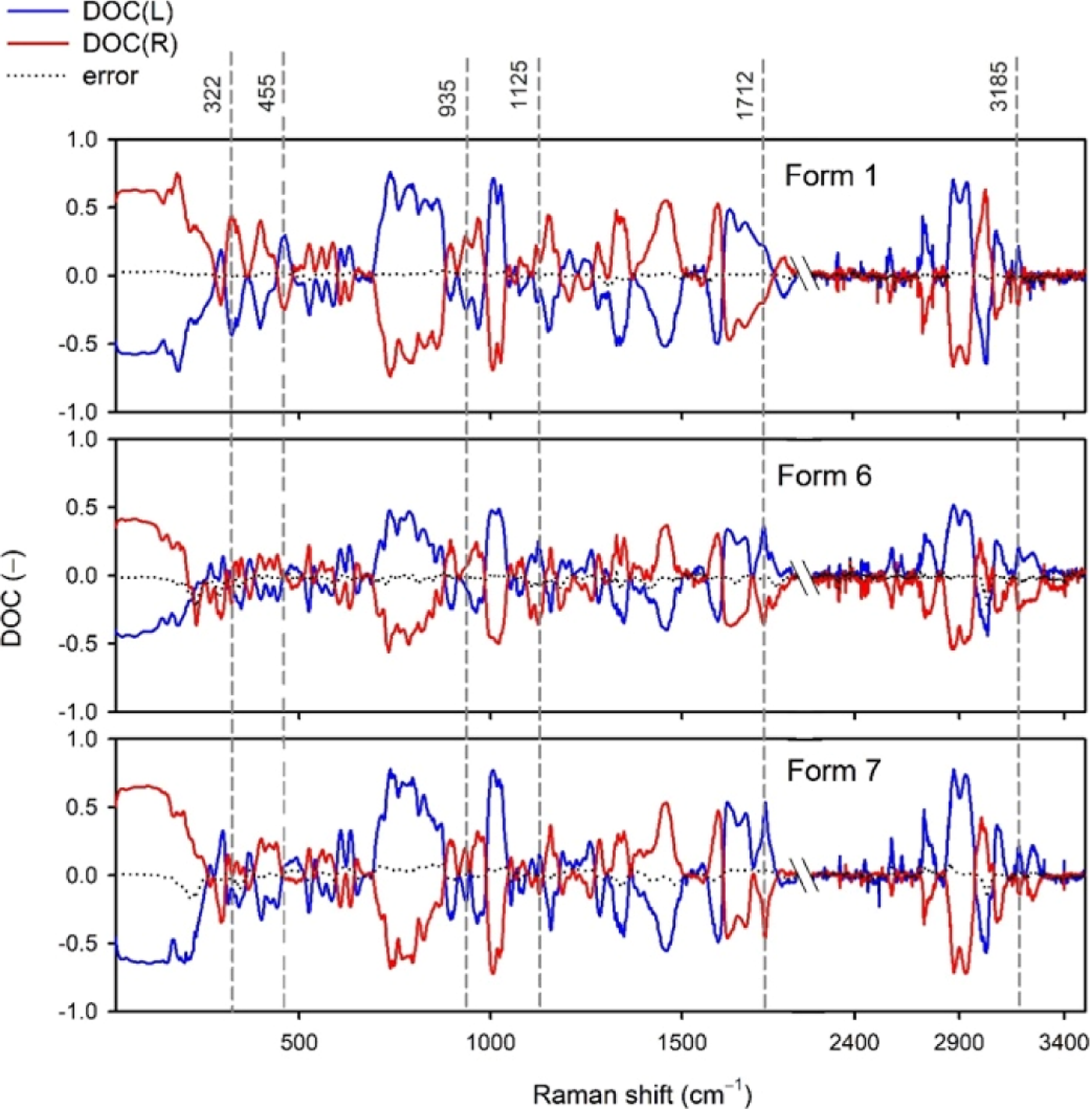
Experimental DOC ratios of sofosbuvir forms. The most prominent differences are marked with vertical dashed lines. The region between 1800 and 2200 cm^–1^ is omitted.

Our experimental results indicate that it is possible to differentiate polymorphs based on their DOC ratios (Figure 10). For example, the DOC band at 322 cm^–1^ of form 1 is approximately three times more intense than those of form 6 and form 7. Similarly, the DOC(L) band at 455 cm^–1^ is positive for form 1 whereas for form 6 and form 7 the DOC is zero. Form 6 has the lowest values of DOC among the three forms. This correlates with the depolarization ratios, as large DOCs correspond to low ρ and form 6 has the highest ρ values. In Table SV we compare DOC(R) values calculated from ρ_Exp._ for six bands with experimental DOC(R) values, which indicates a good correlation with an error of 0–4%.

The DOC ratios make the distinction possible even for forms 6 and 7, which exhibit almost identical unpolarized and similar linearly polarized Raman spectra. For instance, DOC(R) at 935 cm^–1^ is positive for each form but varies in intensity and shape. DOC(R) at 1125 cm^–1^ is even more specific, as it is positive for form 1 and negative for form 6 and form 7. Moreover, the 1125 cm^–1^ DOC of form 6 is approximately twice as intense as in the case of form 7. The DOC at ∼1712 cm^–1^ has a broad profile for form 1 and is sharp for forms 6 and 7; form 6 additionally exhibits a shoulder close to the band at 1712 cm^–1^ at a higher wavenumber that is not present for forms 1 and 7. Similarly, the DOC profile varies for the individual forms around 3185 cm^–1^. Thus, the 935/1125/1712/3185 cm^–1^ DOC markers appear quite helpful in differentiating between the polymorphs.

The raw *I*_L_^L^, *I*_R_^L^, (*I*_L_^L^ + *I*_R_^L^) and (*I*_R_^L^ – *I*_L_^L^) intensities are provided in Figure SV. *I*_R_^R^ and *I*_L_^R^ spectra are not included, the reason being that they are almost identical to the *I*_L_^L^ and *I*_R_^L^ counterparts. Calculated (PW, zero phonon mode) DOC spectra are presented in Figure SVI.

### Effect of Scanned Area

To confirm that the Raman signal was averaged properly, we obtained the difference (*I*_R_^L^ – *I*_L_^L^) spectra of form 1 under different conditions. We scanned three regions of different size (300 × 300 μm, 100 × 100 μm and 10 × 10 μm) for two ratios of sofosbuvir to KBr (1:1 and 1:100). As seen in Figure SVII, the average (*I*_R_^L^ – *I*_L_^L^) spectrum acquired over a 100 × 100 μm scan area from pellets containing a 1:1 ratio of sofosbuvir to KBr matched that acquired across a 300 × 300 μm scan area. However, the spectrum obtained from 1:100 pellets over a 300 × 300 μm scan area had different relative intensities. Therefore, even for a large scanned area, the low API concentration did not provide reliable spectra. The spatial/area anisotropy was even more pronounced for form 6 (Figure SVIII), which inherently forms the largest crystals.^20^ They are spread across the scanned area, and the (*I*_R_^L^ – *I*_L_^L^) spectrum from 1:100 pellets and a 300 × 300 μm scan area did not match the spectral pattern or relative intensities observed for more concentrated samples.

### Spectral Similarity Indices of Experimental LPRS and CPRS Spectra

**Figure 11:**
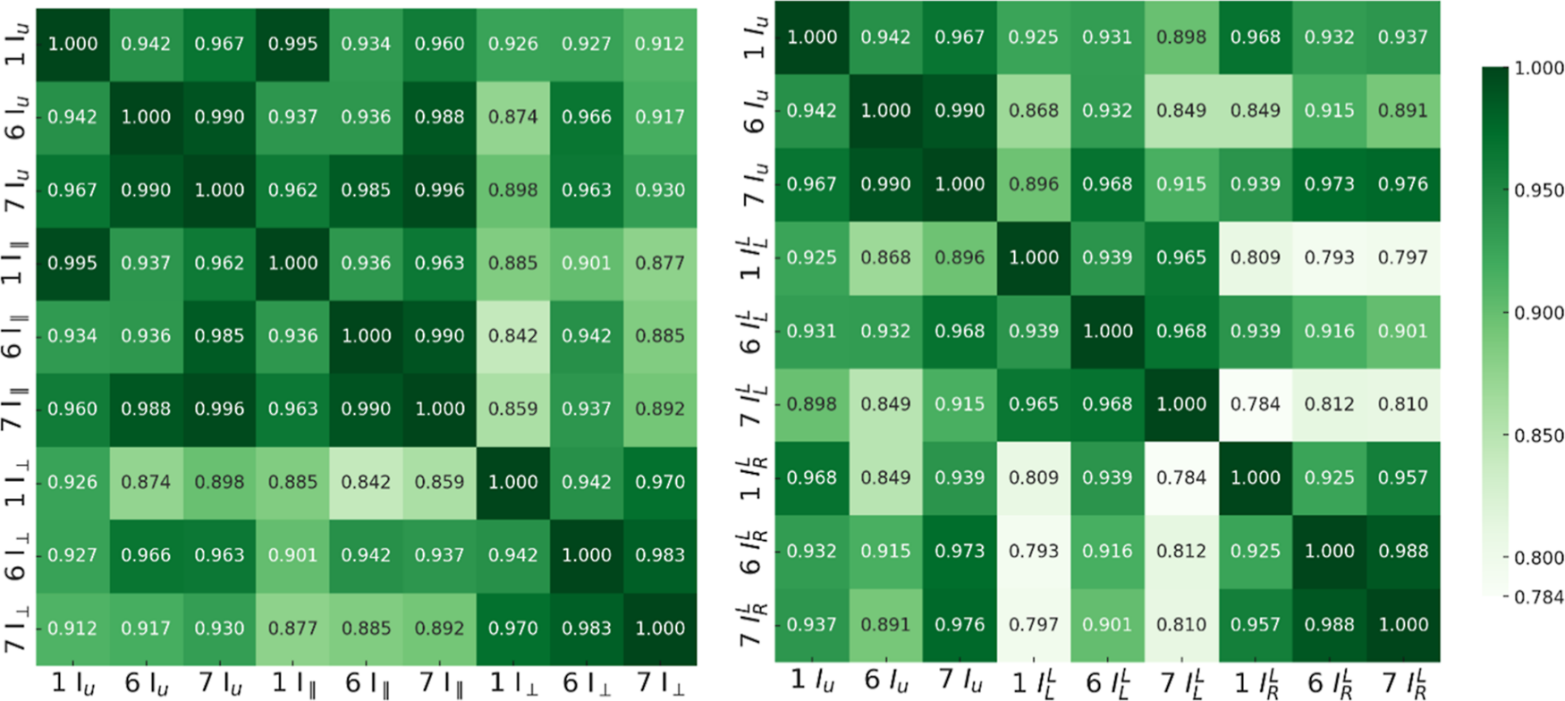
“Heat maps” of spectral similarity indices between experimental LPRS (left) and CPRS (right) spectra. The indices are calculated for integrals within the range of 200–1800 cm^–1^.

For an objective comparison, we calculated spectral similarity indices (σ)^70^ in the 200–1800 cm^–1^ region (Figure 11) as
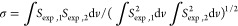


For the same spectra, σ = 1, smaller values indicate disagreement. In LPRS, for *I*_⊥_ σ vary by ∼2 to 6% among the three forms whereas for *I*_∥_, σ vary by 1–6%. As expected, the highest values were obtained between the unpolarized spectra of forms 6 and 7, σ = 0.990. Comparison between form 6 and form 7 yields a value of 0.983. Using this indicator, in the fingerprint region, the *I*_⊥_ spectra can differentiate between form 6 and form 7 about twice as efficiently as unpolarized spectra. CPRS offers even better distinction power than LPRS. The *I*_L_^L^ spectra differ by ∼3 to 6% and the *I*_R_^L^ spectra differ by ∼4 to 7%. For forms 6 and 7, , which is three times greater compared to σ of unpolarized spectra. This makes the (*I*_R_^R^ – *I*_L_^R^), (*I*_R_^L^ – *I*_L_^L^) spectra DOCs convenient for polymorph discrimination, as the bisignate character of these spectra better highlights variations among chemical species. This could, for example, facilitate polymorph differentiation in industrial quality control.

The LPRS spectra in the LF region (20–200 cm^–1^, Figure SIX) made the distinction even more reliable, as the unpolarized spectra between form 6 and form 7 give σ_*I*_u__ = 0.953 whereas the parallel ones give σ_*I*_∥__ = 0.932. Nevertheless, the σs calculated for the stretching region (2700–3400 cm^–1^, see Figure SX for LPRS and Figure SXI for CPRS) are similar to values for the fingerprint region.

## Conclusion

To our knowledge, this study is the first to demonstrate the potential of LPRS and CPRS for differentiating drug polymorphs. We investigated the efficiency of unpolarized, linearly polarized and circularly polarized Raman spectra at resolving polycrystalline sofosbuvir forms 1, 6, and 7 in a broad spectral region (20–3500 cm^–1^). By calculating the spectra at the DFT level, we were able to assign the main spectral features to their corresponding vibrational modes, including LF ones.

Although the unpolarized Raman spectra of forms 6 and 7 were nearly indistinguishable in the fingerprint and CH stretching regions, the LF one provided spectral patterns distinct for each form. We observed that the intensities of LF bands were approximately three times greater than for typical fingerprint bands. Based on calculations, we attributed a large part of the intensity enhancement to the vibrations of highly polarizable phenyl and uracil rings.

In contrast to unpolarized Raman spectroscopy, its linearly polarized variant was able to distinguish between forms 6 and 7 even in the fingerprint region and the CH-stretching region. Form 6 exhibited the highest values of ρ_Exp._, forms 1 and 7 gave similar ρ_Exp._ values. CPRS further enhanced the distinction. DOC ratios at 935, 1125, 1712, and 3185 cm^–1^ were proposed as the best markers for distinguishing polymorphs of sofosbuvir.

By calculating spectral similarity indices, we could better quantify the performance of unpolarized, LPRS and CPRS spectra in resolving sofosbuvir polymorphs. For example, in the fingerprint region, LPRS was approximately twice as effective and CPRS three times as effective as the unpolarized spectra at differentiating the structurally similar forms 6 and 7. Both types of polarized Raman spectroscopy can be effectively used in simplified or automated quality control of pharmaceuticals.

## Data Availability

The data sets generated during the current study are available via the OSF data repository (doi: 10.17605/OSF.IO/S45DU; https://osf.io/s45du).

## Supporting Information Available

Additional details of the procedures can be found (PDF)
