# Supplementary material for: Sofosbuvir Polymorphs Distinguished by Linearly and Circularly Polarized Raman Microscopy
Source: Anal Chem. 2024 Nov 21;96(48):18983–93. doi: 10.1021/acs.analchem.4c03573 (PMC11618743; doi:10.1021/acs.analchem.4c03573)
Supplement: Supplementary file 1 — ac4c03573_si_001.pdf [file ac4c03573_si_001.pdf]

Sofosbuvir polymorphs distinguished by linearly and circularly polarized Raman microscopy

Věra Schrenková,[†,‡] Josef Kapitán,[¶] Petr Bouř,[†] Argyro Chatziadi,[‡] Adam Sklenář,[†,‡] and Jakub Kaminský[†,*]

†Institute of Organic Chemistry and Biochemistry of the Academy of Sciences, Flemingovo nám. 2, Prague, 16610, Czech Republic

‡University of Chemistry and Technology Prague, Technická 5, Prague, 16628, Czech Republic

¶Palacký University Olomouc, Křížkovského 511/8, Olomouc, 77900, Czech Republic

E-mail: jakub.kaminsky@uochb.cas.cz

Spectra Simulations.

The input geometries of three sofosbuvir polymorphs were constructed from X-ray structures (CCDC entries: CUZROG01, CUZROG02, CUZROG03) using the program *Mercury*{Macrae, 2020 #44}). Starting from a cluster of 3×3×3 elementary cells, molecular pairs formed by molecules closer than 4 Å were made (our own program *contact*{Bouř, 2023 #69}) for computations of vibrational properties.

Because positions of the hydrogen atoms determined by X-ray may not be exact, we used three computational strategies to refine the geometry. In the first approach, the geometry of the unit cell was optimized using the plane-wave basis sets technique as implemented in the CASTEP{Clark, 2005 #26} program before creating the 3×3×3 fragment. Alternatively, only the molecular pairs detected and created from the fragment were partially optimized using the QGRAD code{Bouř, 2020 #70} linked to Gaussian 16 (ref.{Frisch, 2016 #48}) in normal mode vibrational coordinates.{Bouř, 2002 #47} In the third "combined" approach, the crystal unit cell was pre-optimized in CASTEP, and then the molecular pairs were optimized using the normal mode coordinates. For the CASTEP, we used the OTFG norm-conserving pseudopotentials for core electrons, 990 eV energy cut-off, ultra-fine SCF, the PBE functional{Perdew, 1996 #51} with the G06 dispersion correction{Grimme, 2006 #52} and the Brillouin zone fine k-point setup of 1×2×1. The lattice size was fixed. For the normal mode optimization, frequencies greater than 100 cm^{-1} were relaxed{Bouř, 2002 #47}, and we used the B3LYP/6-311++G(d,p) level of theory.

The harmonic force field and polarizability derivatives were then calculated for the optimized molecular pairs using the Gaussian program{Frisch, 2016 #48} at the B3LYP/6-311++G(d,p) level. The crystal environment was simulated by the CPCM solvent model{Takano, 2005 #54} and relative permittivity $\varepsilon_r = 78$.{Dračínský, 2013 #25} Subsequently, the atomic property tensors

were transferred back to the 3×3×3 cluster using the Cartesian coordinate transfer scheme (*cctn* program{Bouř, 1997 #49}). Vibrational frequencies and Raman intensities were calculated in two ways: 1) directly for the 3×3×3 cluster, and 2) using the dynamic matrix constructed from the same force field and the zero (0, 0, 0) crystal phonon modes. Line intensities, depolarization and DOC ratios were convoluted with Lorentzian bands of 10 cm^{-1} full width at half-height.

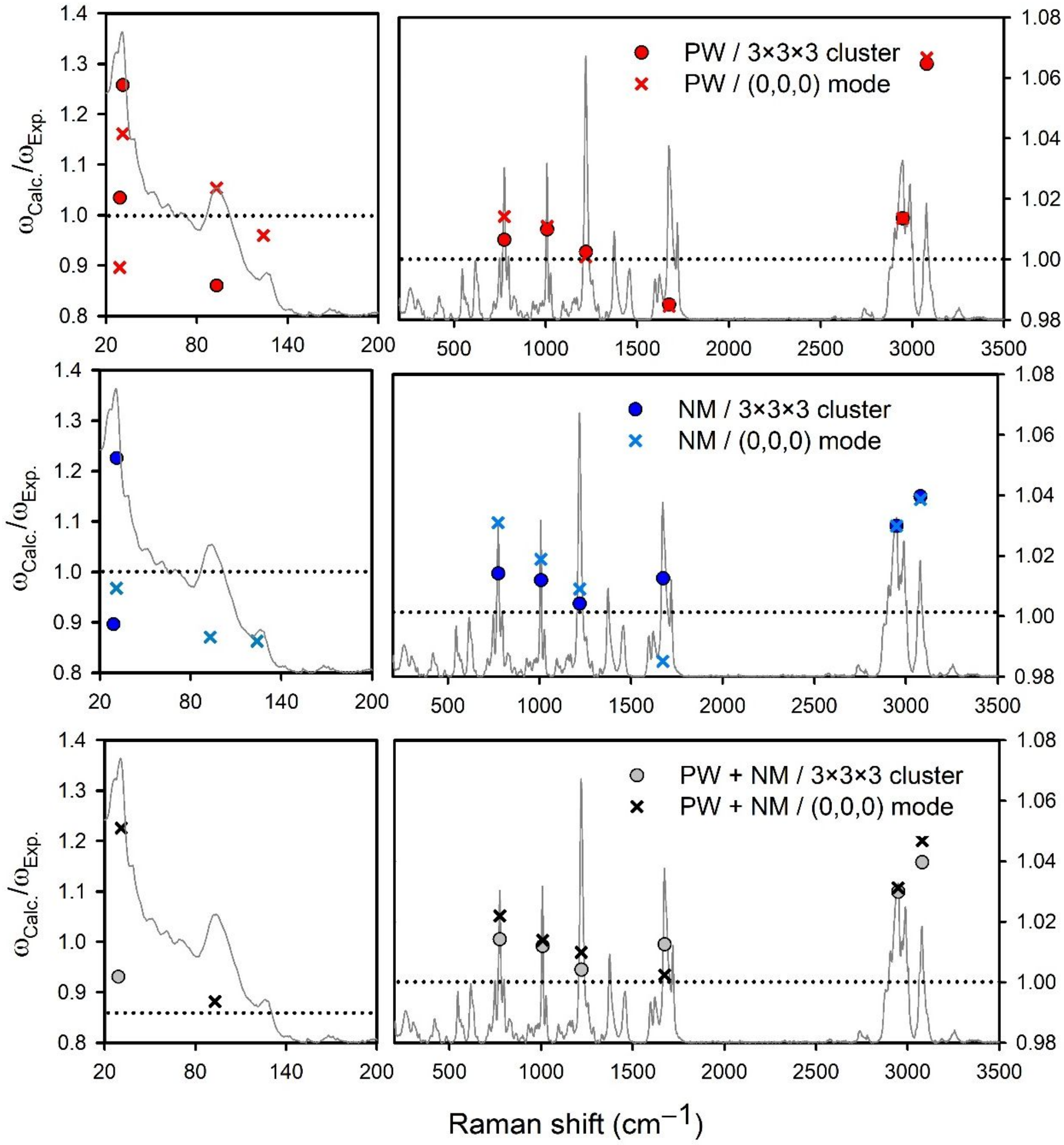

Figure SI Ratios of calculated to experimental frequencies ($\omega_{Calc.}/\omega_{Exp.}$) for the three calculational approaches (PW, NM, PW+NM). Zero phonon mode and 3×3×3 cluster spectra are compared.

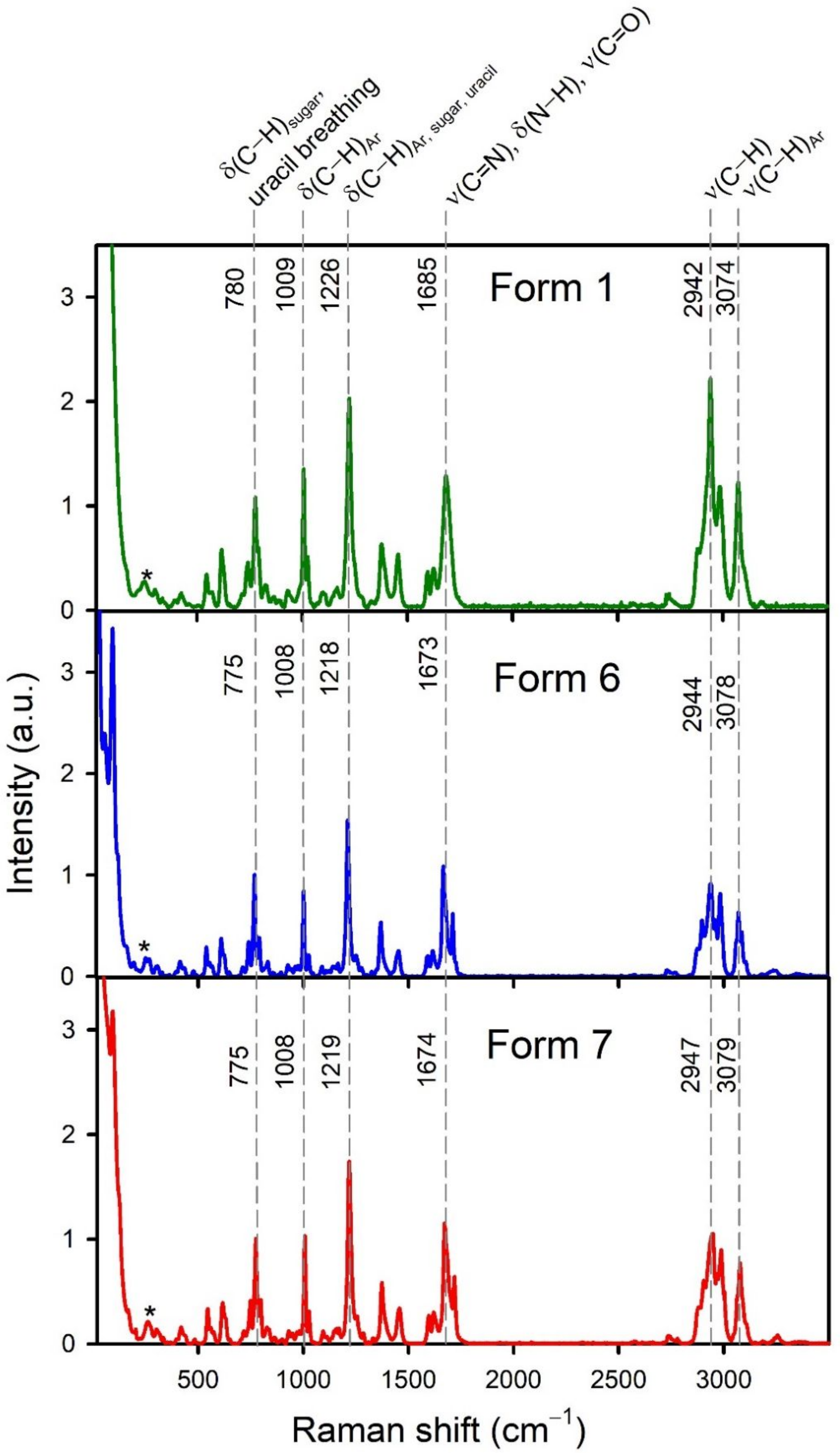

Figure SII Unpolarized experimental Raman spectra of sofosbuvir form 1, 6 and 7 in the full spectral region. The spectra are normalized to the ~775 cm^{-1} band. Asterisk marks KBr (matrix) Raman band.

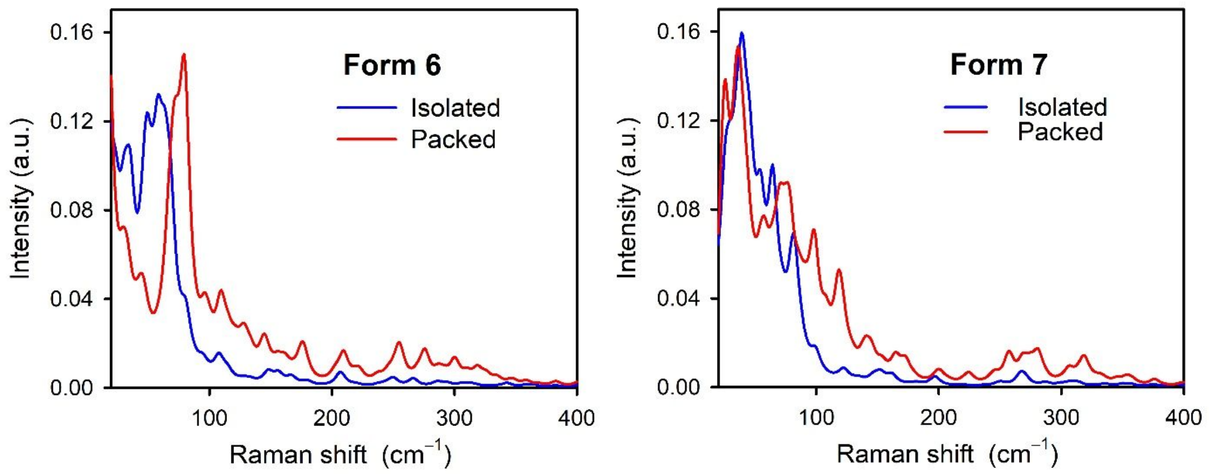

Figure SIII Calculated unpolarized Raman spectra of sofosbuvir form 6 and 7 – isolated molecule in vacuum (blue) and packed in crystal (red). Geometry of isolated sofosbuvir was taken from PW optimized unit cell. Spectra of packed forms are normalized to number of molecules in elementary cell.

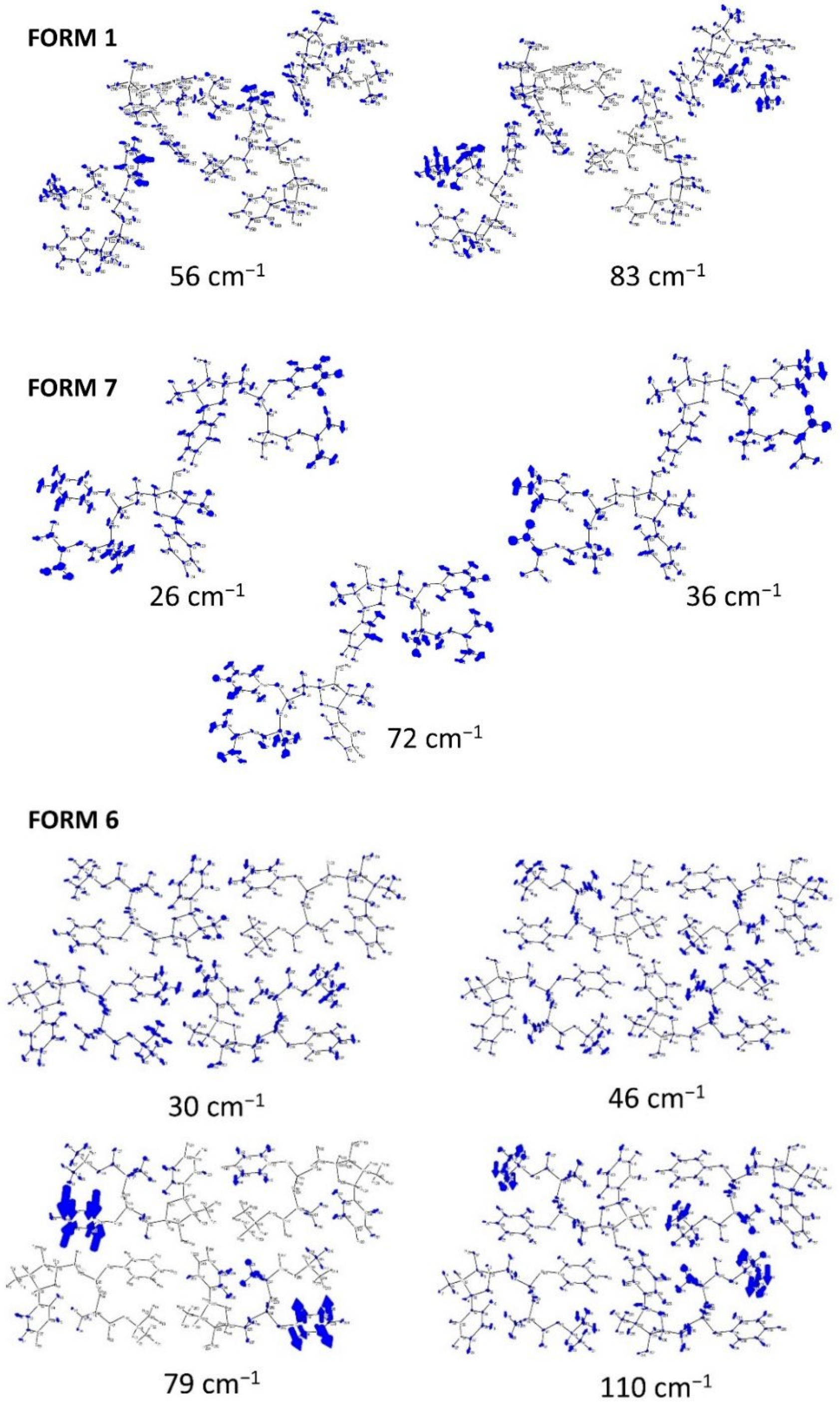

Figure SIV Visualization of calculated (PW, zero phonon mode) low-frequency vibrational modes.

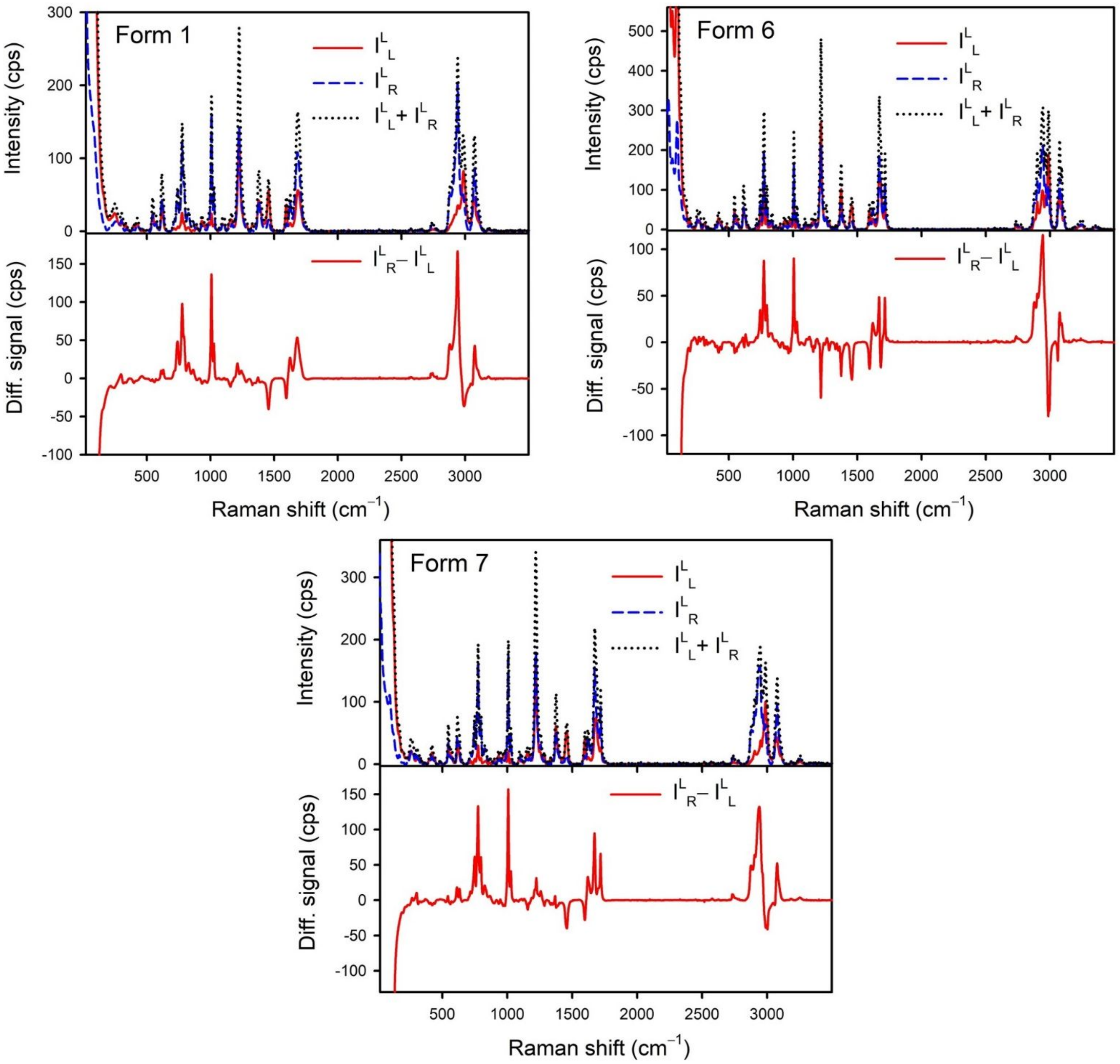

Figure SV Experimental I_L^L, I_R^L, sum ($I_L^L + I_R^L$) and $difference$ ($I_R^L - I_L^L$) Raman spectra of sofosbuvir form 1, 6 and 7.

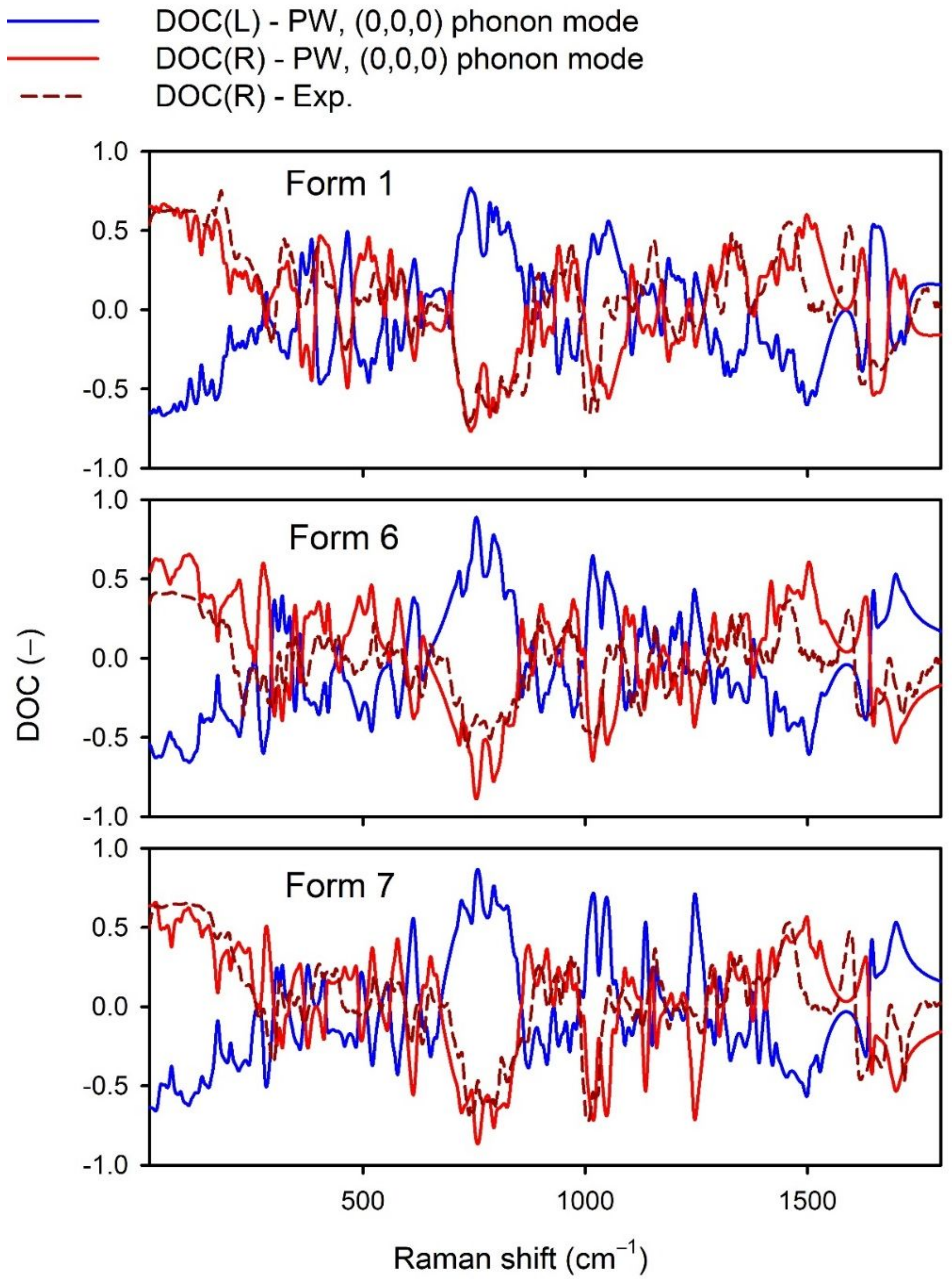

Figure SVI Comparison of calculated (PW, zero phonon mode) DOC spectra with experimental DOC(R).

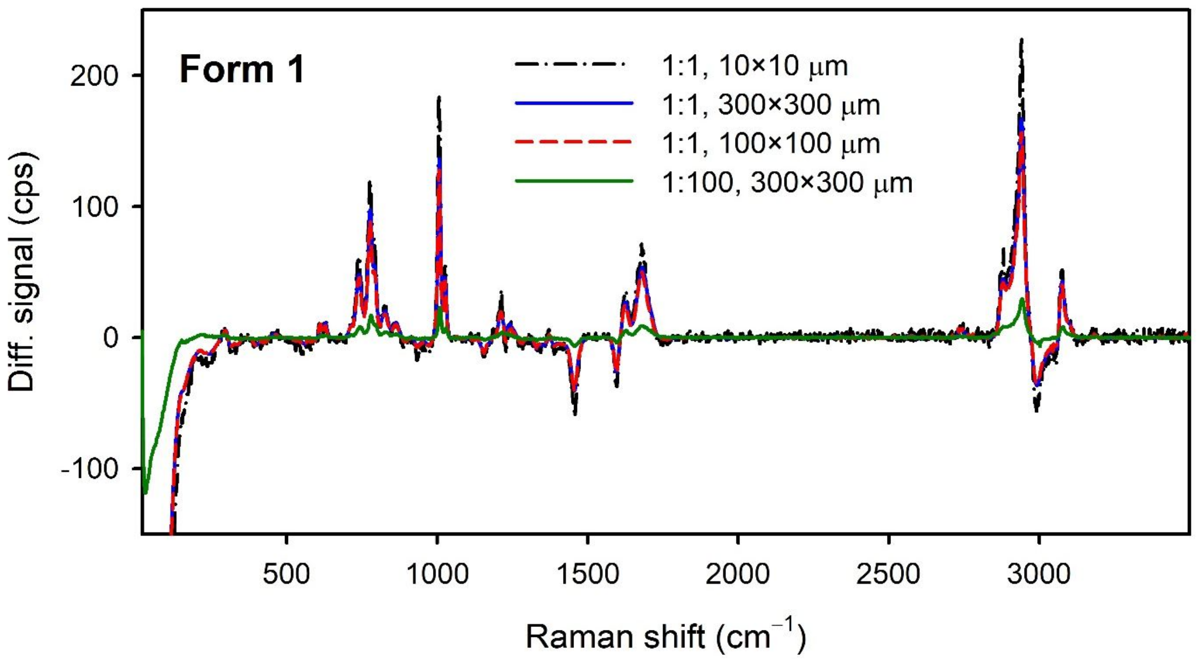

Figure SVII Comparison of difference ($I_R^L - I_L^L$) spectra of form 1 obtained with different experimental conditions (scanned area of 300×300/100×100/10×10 μm, sofosbuvir to KBr ratio 1:1 or 1:100).

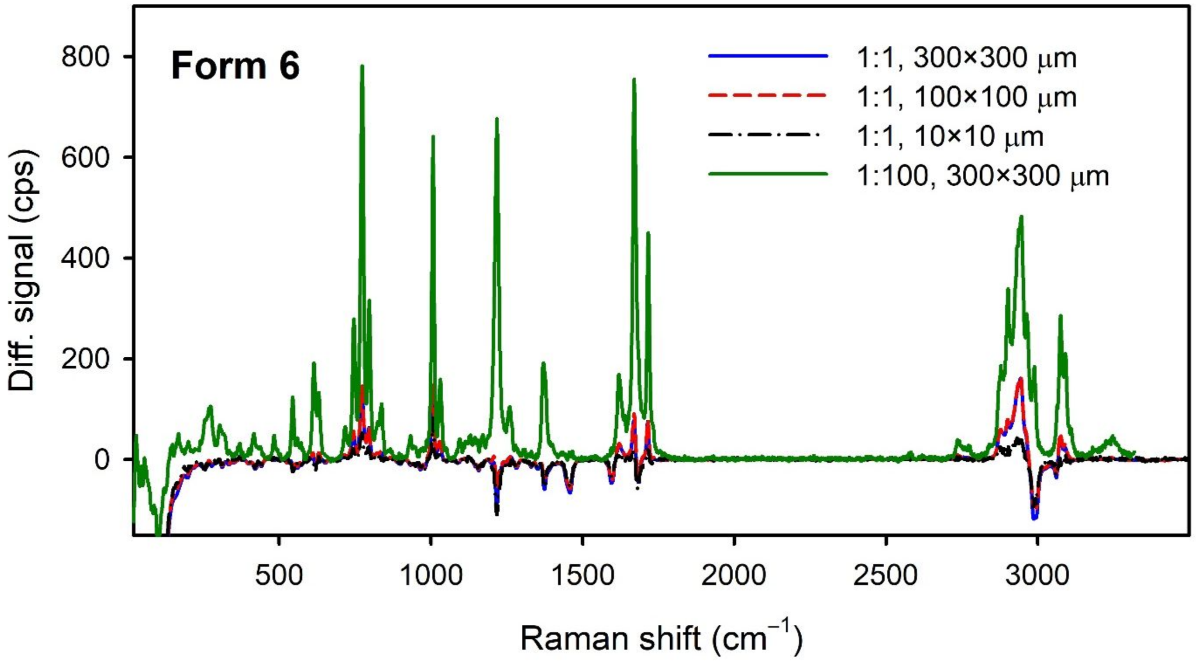

Figure SVIII Comparison of difference ($I_R^L - I_L^L$) spectra of form 6 obtained with different experimental conditions (scanned area of 300×300/100×100/10×10 μm, sofosbuvir to KBr ratio 1:1 or 1:100).

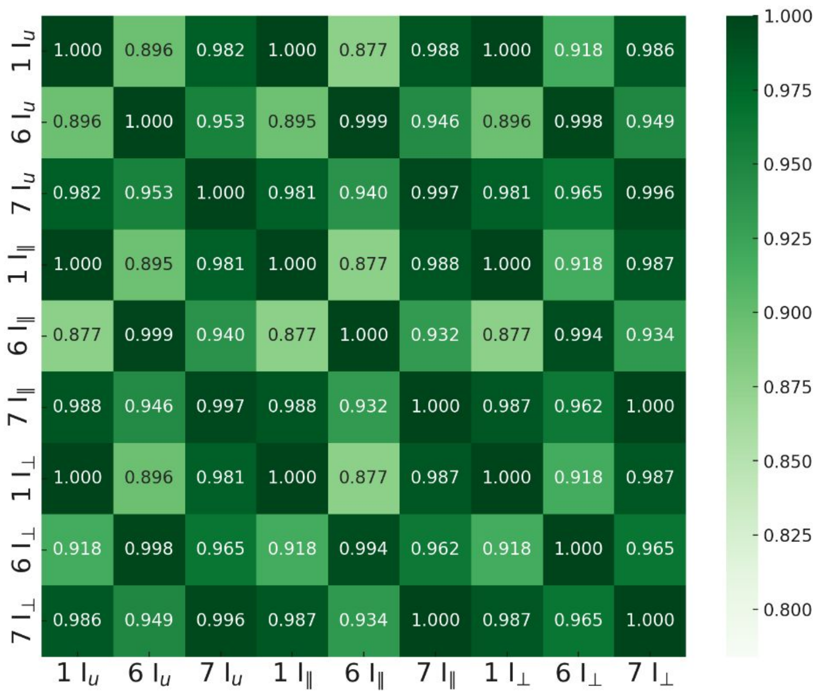

Figure SIX Heat maps of spectral similarity indices between experimental LPRS spectra. The indices are calculated for the integrals within 20-200 cm^{-1}, the spectra were recorded with 1800 g/mm spectral grating.

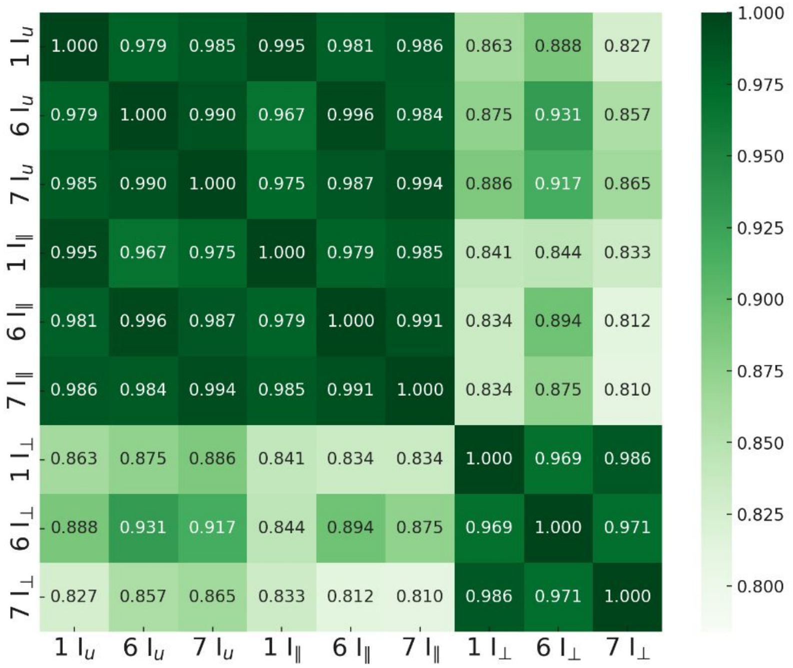

Figure SX Heat maps of spectral similarity indices between experimental LPRS spectra. The indices are calculated for the integrals within 2700-3400 cm^{-1}, the spectra were recorded with 600 g/mm spectral grating.

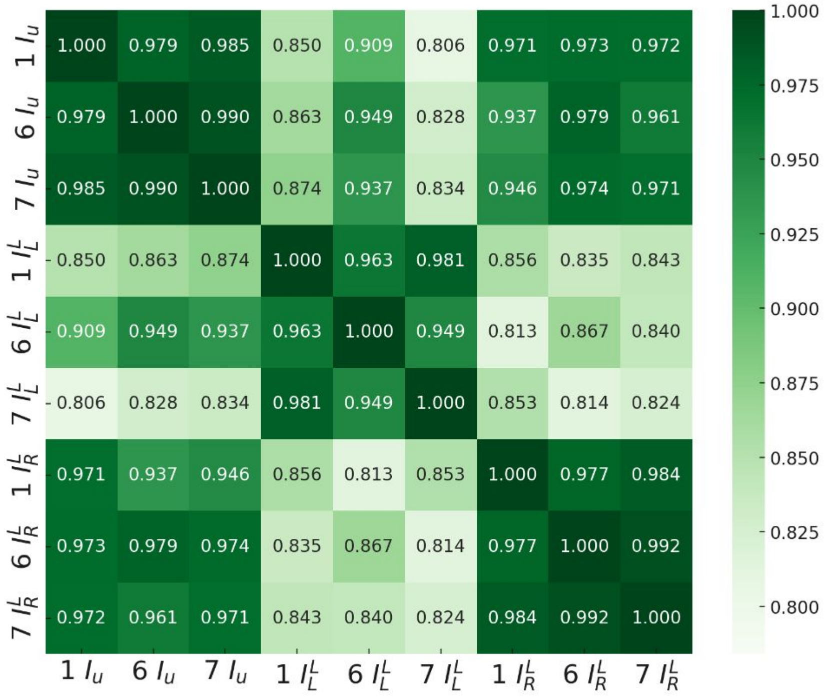

Figure SXI Heat maps of spectral similarity indices between experimental CPRS spectra. The indices are calculated for the integrals within 2700-3400 cm^{-1}, the spectra were recorded with 600 g/mm spectral grating.

Table SI. Band assignments and depolarization ratios for sofosbuvir form 1, 6 and 7. Experimental frequencies and depolarization ratios <200 cm^{-1} are obtained from spectra recorded with 1800 g/mm grating, otherwise with 600 g/mm grating. Calculated frequencies (cm^{-1}) and depolarization ratios are obtained from spectra calculated using the PW approach, zero (0,0,0) phonon mode.

**Form 1**				**Form 6**				**Form 7**				
**Exp.**	$\rho_{Exp.}$	**Calc.**	$\rho_{Calc.}$	**Exp.**	$\rho_{Exp.}$	**Calc.**	$\rho_{Calc.}$	**Exp.**	$\rho_{Exp.}$	**Calc.**	$\rho_{Calc.}$	**Assignment**
–	–	35	0.70	28	0.72	30	0.68	29	0.76	26	0.70	
59	0.66	56	0.71	36	0.74	46	0.68	31	0.76	36	0.70	
–	–	67	0.70	100	0.70	79	0.63	93	0.78	72	0.65	
83	0.61	83	0.69	125	0.80	110	0.68	124	0.77	119	0.64	
780	0.09	785	0.12	775	0.17	794	0.06	775	0.10	796	0.06	δ(C–H)$_{sugar}$, uracil deformation
1009	0.07	1018	0.15	1008	0.14	1019	0.08	1008	0.07	1019	0.08	δ(C–H)$_{Ar}$
1226	0.32	1227	0.32	1218	0.37	1225	0.36	1219	0.32	1220	0.37	δ(C–H)$_{sugar}$, δ(C–H)$_{uracil}$, δ(C–H)$_{Ar}$
1685	0.19	1647	0.13	1673	0.25	1651	0.24	1674	0.19	1648	0.19	ν(C=N), δ(N–H)$_{sec.}$ ν(C=O)
2942	0.08	2996	0.07	2944	0.16	2998	0.05	2947	0.10	2988	0.03	ν(C–H)$_s$
3074	0.20	3158	0.19	3078	0.24	3162	0.20	3079	0.19	3162	0.20	ν(C–H)$_{s,\ Ar}$

Table SII. Calculated (PW approach) frequencies (cm^{-1}) and depolarization ratios for six selected bands in the fingerprint and hydrogen stretching region (200-3500 cm^{-1}).

**Form 1**				**Form 6**				**Form 7**			
PW / (0,0,0) phonon mode		PW / 3×3×3 crystal		PW / (0,0,0) phonon mode		PW / 3×3×3 crystal		PW / (0,0,0) phonon mode		PW / 3×3×3 crystal	
Calc.	$\rho_{calc.}$	Calc.	$\rho_{calc.}$	Calc.	$\rho_{calc.}$	Calc.	$\rho_{calc.}$	Calc.	$\rho_{calc.}$	Calc.	$\rho_{calc.}$
785	0.12	782	0.11	794	0.06	791	0.09	796	0.06	780	0.12
1018	0.15	1017	0.13	1019	0.08	1018	0.09	1019	0.08	1018	0.09
1227	0.32	1230	0.33	1225	0.36	1227	0.38	1220	0.37	1222	0.33
1647	0.13	1645	0.17	1651	0.24	1653	0.17	1648	0.19	1649	0.17
2996	0.07	2993	0.07	2998	0.05	2997	0.06	2988	0.03	2988	0.03
3158	0.19	3151	0.18	3162	0.20	3157	0.17	3162	0.20	3155	0.16

Table SIII. Calculated (NM approach) frequencies (cm^{-1}) and depolarization ratios for six selected bands in the fingerprint and hydrogen stretching region (200-3500 cm^{-1}).

**Form 1**				**Form 6**				**Form 7**			
NM / (0,0,0) phonon mode		NM / 3×3×3 crystal		NM / (0,0,0) phonon mode		NM / 3×3×3 crystal		NM / (0,0,0) phonon mode		NM / 3×3×3 crystal	
Calc.	$\rho_{calc.}$	Calc.	$\rho_{calc.}$	Calc.	$\rho_{calc.}$	Calc.	$\rho_{calc.}$	Calc.	$\rho_{calc.}$	Calc.	$\rho_{calc.}$
787	0.07	785	0.09	789	0.08	787	0.08	799	0.06	784	0.12
1020	0.14	1019	0.11	1019	0.09	1020	0.08	1027	0.06	1019	0.07
1224	0.22	1223	0.27	1230	0.28	1228	0.32	1230	0.36	1232	0.32
1698	0.22	1694	0.29	1679	0.25	1696	0.33	1649	0.34	1694	0.33
3044	0.11	3042	0.05	3048	0.08	3033	0.08	3036	0.04	3042	0.06
3209	0.20	3196	0.19	3207	0.17	3198	0.22	3198	0.21	3195	0.27

Table SIV. Calculated (PW + NM approach) frequencies (cm^{-1}) and depolarization ratios for six selected bands in the fingerprint and hydrogen stretching region (200-3500 cm^{-1}).

**Form 1**				**Form 6**				**Form 7**			
NM + PW / (0,0,0) phonon mode		NM + PW / 3×3×3 crystal		NM + PW / (0,0,0) phonon mode		NM + PW / 3×3×3 crystal		NM + PW / (0,0,0) phonon mode		NM + PW / 3×3×3 crystal	
Calc.	$\rho_{calc.}$	Calc.	$\rho_{calc.}$	Calc.	$\rho_{calc.}$	Calc.	$\rho_{calc.}$	Calc.	$\rho_{calc.}$	Calc.	$\rho_{calc.}$
789	0.07	786	0.10	788	0.15	785	0.07	792	0.06	786	0.13
1021	0.13	1020	0.10	1022	0.07	1021	0.07	1022	0.07	1020	0.08
1227	0.26	1221	0.27	1235	0.29	1228	0.33	1231	0.32	1224	0.31
1698	0.22	1697	0.29	1680	0.25	1699	0.34	1678	0.28	1695	0.33
3045	0.14	3039	0.04	3056	0.06	3037	0.05	3040	0.04	3036	0.08
3213	0.21	3198	0.18	3216	0.20	3200	0.18	3223	0.22	3201	0.17

Table SV ρ values calculated from experimental DOC(R), directly measured $\rho_{Exp.}$ and error (%).

**Raman shift / cm^{-1}**	**Form 1**			**Form 6**			**Form 7**		
	ρ	$\rho_{Exp.}$	Error	ρ	$\rho_{Exp.}$	Error	ρ	$\rho_{Exp.}$	Error
775	0.11	0.09	2	0.18	0.17	1	0.11	0.10	1
1008	0.09	0.07	2	0.15	0.14	1	0.08	0.07	1
1218	0.34	0.32	2	0.33	0.37	4	0.34	0.32	2
1673	0.20	0.19	1	0.24	0.25	1	0.21	0.19	2
2944	0.10	0.08	2	0.16	0.16	0	0.11	0.10	1
3078	0.21	0.20	1	0.23	0.24	1	0.2	0.19	1
